# A 5-µm-thick electrolyte with a high-entropy interphase for practical solid-state lithium metal batteries

**DOI:** 10.1093/nsr/nwag511

**Published:** 2026-08-25

**Authors:** Xuexia Lan, Yi Zeng, Ye Chen, Zhengjie Chen, Zhen Li, Xinyuan Hu, Ziyong Li, Qiutan Liu, Renzong Hu, Jing Peng, Hui-Ming Cheng

**Affiliations:** Shenzhen Key Laboratory of Energy Materials for Carbon Neutrality, Institute of Technology for Carbon Neutrality, Shenzhen Institutes of Advanced Technology, Chinese Academy of Sciences, Shenzhen 518055, China; Faculty of Materials Science and Energy Engineering, Shenzhen University of Advanced Technology, Shenzhen 518107, China; School of Materials Science and Engineering, Hefei University of Technology, Hefei 230009, China; Shenzhen Key Laboratory of Energy Materials for Carbon Neutrality, Institute of Technology for Carbon Neutrality, Shenzhen Institutes of Advanced Technology, Chinese Academy of Sciences, Shenzhen 518055, China; Faculty of Materials Science and Energy Engineering, Shenzhen University of Advanced Technology, Shenzhen 518107, China; Faculty of Materials Science and Energy Engineering, Shenzhen University of Advanced Technology, Shenzhen 518107, China; Shenzhen Key Laboratory of Energy Materials for Carbon Neutrality, Institute of Technology for Carbon Neutrality, Shenzhen Institutes of Advanced Technology, Chinese Academy of Sciences, Shenzhen 518055, China; School of Materials Science and Engineering, Guangdong Provincial Key Laboratory of Advanced Energy Storage Materials, South China University of Technology, Guangzhou 510640, China; Department of Chemical Engineering, University College London, London WC1E 7JE, UK; School of Materials Science and Engineering, Guangdong Provincial Key Laboratory of Advanced Energy Storage Materials, South China University of Technology, Guangzhou 510640, China; Shenzhen Key Laboratory of Energy Materials for Carbon Neutrality, Institute of Technology for Carbon Neutrality, Shenzhen Institutes of Advanced Technology, Chinese Academy of Sciences, Shenzhen 518055, China; School of Materials Science and Engineering, Guangdong Provincial Key Laboratory of Advanced Energy Storage Materials, South China University of Technology, Guangzhou 510640, China; Shenzhen Key Laboratory of Energy Materials for Carbon Neutrality, Institute of Technology for Carbon Neutrality, Shenzhen Institutes of Advanced Technology, Chinese Academy of Sciences, Shenzhen 518055, China; Faculty of Materials Science and Energy Engineering, Shenzhen University of Advanced Technology, Shenzhen 518107, China; State Key Laboratory of Precision and Intelligent Chemistry, University of Science & Technology of China, Hefei 230026, China; Shenzhen Key Laboratory of Energy Materials for Carbon Neutrality, Institute of Technology for Carbon Neutrality, Shenzhen Institutes of Advanced Technology, Chinese Academy of Sciences, Shenzhen 518055, China; SKKU Global Research Center, Sungkyunkwan University, Suwon 16419, Republic of Korea; Shenyang National Laboratory for Materials Science, Institute of Metal Research, Chinese Academy of Sciences, Shenyang 110016, China

**Keywords:** ultrathin electrolyte, solid–electrolyte interphase, high entropy, current density, energy density

## Abstract

Solid-state lithium metal batteries with ultrathin and lightweight electrolytes are promising for high-energy-density storage but are prone to dendrite penetration under practical current densities. This limitation arises primarily from the uncontrolled and chemically heterogeneous solid–electrolyte interphase (SEI), inducing obvious interfacial instability and leading to uneven and sluggish Li^+^ flux at the interface. Here, we design a homogeneous interphase with high configurational entropy, with its optimized composition screened by machine learning, providing dual functions by stabilizing the SEI structure and promoting efficient Li^+^ transport. The high-entropy nature of this interphase is experimentally supported and promotes uniform and fast interfacial Li^+^ flux, enabling a 5-µm-thick electrolyte to sustain a critical current density of 5.2 mA cm^−2^ and maintain stable long-term lithium plating/stripping at 3 mA cm^−2^ for 850 h. Li||LiNi_0.8_Co_0.1_Mn_0.1_O_2_ pouch cells with this ultrathin and lightweight electrolyte deliver an areal capacity of 5.4 mAh cm^−2^ and an energy density of 573 Wh kg^−1^, showing great potential for practical solid-state lithium metal batteries.

## Teaser

This study presents a high-entropy interphase that enables fast and uniform interfacial Li^+^ flux, allowing a 5-µm-thick electrolyte to resist dendrite penetration and support long-term cycling at practical current densities.

## INTRODUCTION

Solid-state lithium metal batteries (SSLMBs), using solid Li^+^ conductors instead of flammable liquid electrolytes, are considered as a promising route for addressing the energy density and safety limitations of conventional liquid systems [1–4]. Reducing the thickness of solid electrolytes is an effective strategy to decrease the fraction of inactive components and thereby increase cell-level energy density, particularly for low-density polymer electrolytes [5,6]. For example, by thinning the polymer electrolytes to 20 µm, an energy density exceeding 400 Wh kg^−1^ is achievable [7,8]. As ionic conductance scales inversely with electrolyte thickness [9], ultrathin electrolytes mitigate the limitations of insufficient ionic conductivity in some electrolyte systems [8,10], by shortening the Li^+^ transport distance and reducing internal resistance. However, SSLMBs with ultrathin electrolytes remain unable to operate reliably under practical conditions, such as a current density of 3 mA cm^−2^ and an areal capacity of 3 mAh cm^−2^ [11,12]. The primary limitation arises from internal short circuiting caused by lithium dendrite penetration through ultrathin electrolytes.

A dominant mechanistic origin of dendrite formation is a spatially uneven Li^+^ flux at the Liǀelectrolyte interface, which drives localized lithium deposition and subsequent dendritic growth. This uneven flux originates from the formation of an unstable solid–electrolyte interphase (SEI) layer with chemically heterogeneous components, such as LiF, Li_2_S, Li_2_O, Li_3_N and Li_3_P [8,13]. These components have large disparities in ionic conductivity and structural stability, causing pronounced interface-potential fluctuations and inducing localized Li^+^ flux concentration [14,15]. Such irregular Li^+^ distribution promotes dendrite growth and penetration through ultrathin electrolytes, even at a low current

density (≤1 mA cm^−2^) [16–18]. To address these challenges, extensive efforts have focused on electrolyte engineering [4], interfacial regulation [12], artificial SEI construction [19] and ion-transport modulation [20] for stabilizing lithium metal anodes. Representative strategies include increasing the content of inorganic fillers [9], modifying the interfacial layer with high-entropy alloys and Li-compensation components [21,22], promoting interfacial fluorination [23,24], constructing dual protective layers [23,25,26] and introducing electrochemically robust coating layers to improve SEI stability [27–29]. However, these approaches often increase mechanical brittleness, thereby raising the risk of membrane fracture, or introduce high interfacial Li^+^ nucleation barriers that partially shield Li^+^ flux [17,27]. To enable ultrathin electrolytes to maintain mechanical integrity without compromising interfacial electrochemical performance, an effective interface design strategy is needed to integrate SEI uniformity with rapid Li^+^ transport.

Here, we designed an interfacial region for ultrathin solid electrolytes to enable uniform and fast Li^+^ nucleation and deposition, through an SEI enriched with a homogeneous high-entropy interphase (HEI). This interphase provides dual functionality by stabilizing the SEI structure and facilitating Li^+^ transport. Its composition, screened by machine learning, combines entropy-driven stability with a low Li^+^ diffusion barrier. Experimentally validated by using poly(vinylidene fluoride-co-hexafluoropropylene) (PVDF-HFP) with pronounced interfacial instability as a model system, a 5-µm-thick PVDF-HFP/HEI electrolyte sustains a critical current density of 5.2 mA cm^−2^ and stable lithium plating/stripping at 3 mA cm^−2^ for 850 h. Li||LiNi_0.8_Co_0.1_Mn_0.1_O_2_ cells achieve stable cycling over 1000 cycles at 3 mA cm^−2^, with an average Coulombic efficiency (CE) of 99.95%. Pouch cells with this ultrathin and lightweight electrolyte deliver an areal capacity of 5.4 mAh cm^−2^ and an energy density of 573 Wh kg^−1^. This HEI strategy provides a new avenue for developing high-performance ultrathin electrolytes and practical SSLMBs.

## RESULTS AND DISCUSSION

### Optimized composition screened by machine learning

In a pure PVDF-HFP electrolyte, strong interactions between lithium salts and N,N-dimethylformamide (DMF) solvent form a [Li(DMF)_x_]^+^ solvation structure (Fig. 1a). It is prone to electron transfer with reactive lithium metal, resulting in unstable SEI with multicomponents [14,15]. This interface degradation becomes exacerbated at high current densities, producing uncontrolled and thick SEI [2]. The resulting heterogeneous SEI impedes efficient Li^+^ transport, causes uneven Li^+^ flux and accelerates lithium dendrites growth [30,31]. Constructing an SEI enriched with a homogeneous interphase that combines high stability with fast ionic transport is expected to significantly improve interfacial electrochemical performance at high current densities. Advances in inorganic ionic conductors show that high-entropy strategies can integrate entropy-driven stability with improved ionic migration efficiency [32,33]. For example, high-entropy superionic conductors, including Li_9.54_[Si_0.6_Ge_0.4_]_1.74_P_1.44_S_11.1_B_r0.3_O_0.6_ [32] and Li_5.5_PS_4.5_Cl_0.8_Br_0.7_ [34], achieve ionic conductivities of 32 and 22.2 mS cm^−1^ at 25°C, revealing the role of anion-sublattice disorder in promoting Li^+^ transport [35,36].

**Figure 1.**
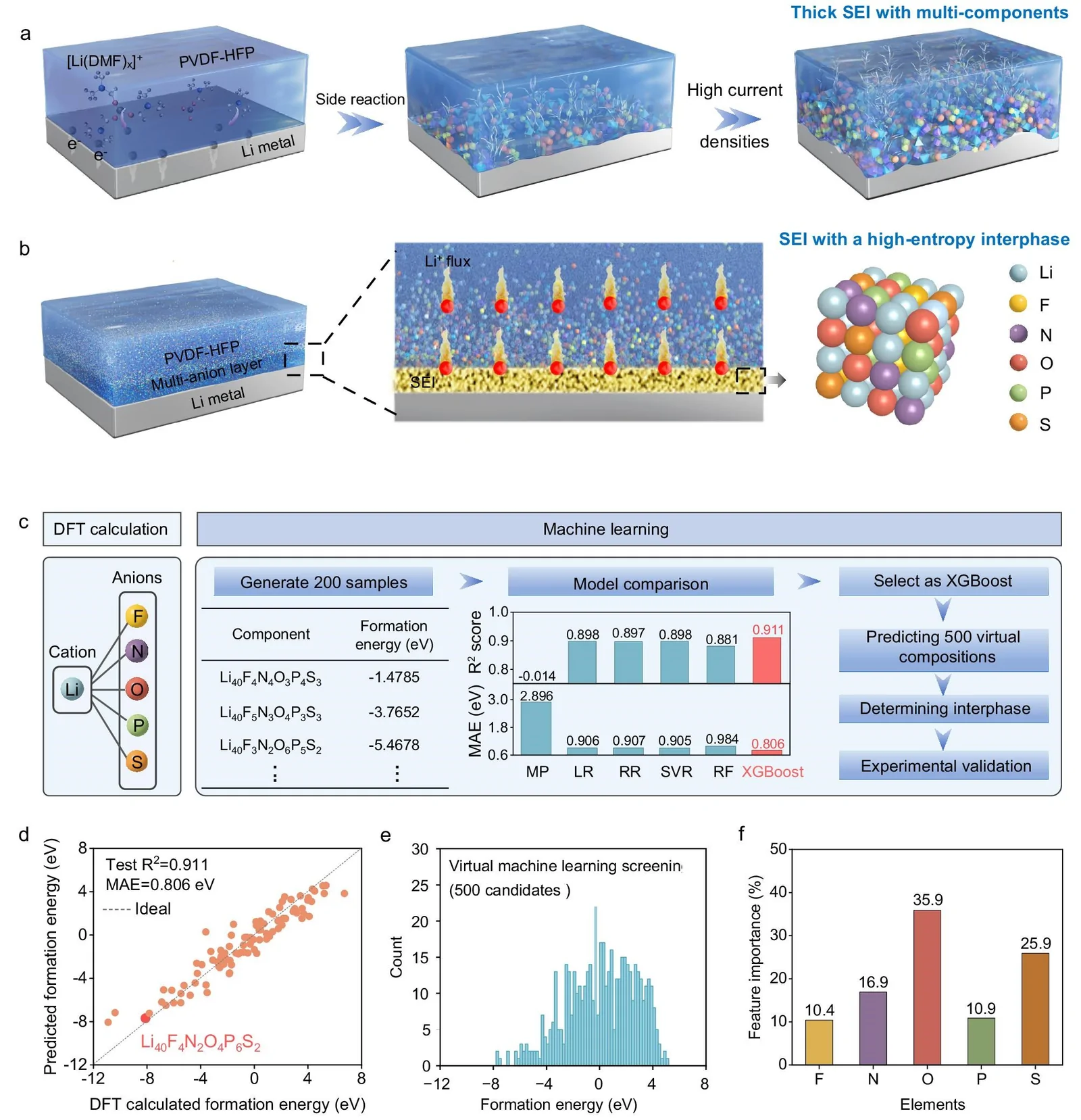
Interfacial reactions between lithium metal and PVDF-HFP-based electrolytes, and machine-learning-guided design of an HEI. (a) Schematic of interfacial electron transfer. (b) Schematic of an SEI with an HEI. (c) Workflow of the machine-learning-guided strategy for designing an HEI, including the comparison of R^2^ and MAE of test set for different models. (d) Parity plot showing predicted versus DFT-calculated formation energies for the independent test datasets. (e) Distribution of predicted formation energies for 500 virtual Li-F-N-O-P-S compositions screened using the trained XGBoost model. (f) Feature importance analysis showing the relative contribution of each compositional element to the model predictions.

To construct an HEI composed of multi-anions, it is necessary to identify the anion types. Using a typical SEI component LiF as a parent model, due to its chemical stability and mechanical robustness [28], density functional theory (DFT) calculations were performed to compare the formation energies of LiF structures doped with various anions (Fig. S1). The examined anions included B, C, N, O, P, S, Cl and Br. The results show that doping LiF with N, O, P or S has significantly lower formation energies than others, indicating that they are more likely to form a single, highly miscible phase structure. This behavior likely arises from their comparable ionic radii and similar electronegativities [37], which facilitate synergistic interactions that promote the formation of an HEI. As illustrated in Fig. 1b, the Liǀelectrolyte interface region enriched with these anions (F, N, O, P and S) facilitates the generation of an SEI containing an HEI.

Even after identifying the anion species, determining the optimal compositional ratios within the HEI remains challenging due to its complex tunability. Machine learning provides a powerful alternative, allowing the prediction of formation energies and accelerating composition screening. It has accelerated the discovery of electrode and electrolyte materials [38–43], offering valuable guidance for screening HEIs. In this work, a dataset was constructed from DFT calculations of 200 high-entropy electrolytes with varying anion ratios. The normalized fractions of F, N, O, P and S were used as input features, and the formation energy was used as the prediction target for thermodynamic stability. Considering the high computational cost of first-principles calculations for high-entropy systems, the dataset is relatively small compared with large-scale materials databases. Therefore, the models were developed as data-efficient screening tools based on a curated and internally consistent DFT dataset, rather than as universal predictors for all interphase chemistries. This strategy is consistent with recent studies showing that reliable materials screening under data-scarce conditions can be achieved using high-quality datasets, physically meaningful descriptors and rigorous validation protocols [40–42,44].

As shown in Fig. 1c, six predictive models, including a mean predictor, linear regression, ridge regression, support vector regression, random forest and XGBoost, were benchmarked using the coefficient of determination (R^2^) and mean absolute error (MAE) as the main evaluation metrics (Fig. S2), supported by a comparison of root mean squared error (RMSE) values and five-fold cross-validation MAE values (Table S1). Among these models, XGBoost showed the best overall performance on both the validation and independent test datasets. In particular, the independent test set showed an R^2^ value of 0.911 and an MAE of 0.806 eV per formula unit, corresponding to 0.014 eV per atom. This test-set error is below the typical uncertainty of DFT calculations (∼0.05 eV per atom) [40,42], indicating that the model predictions are within the intrinsic accuracy limit of first-principles calculations and suggesting robust generalization with minimal overfitting. Additional deep-learning baselines, including a simple fully connected multilayer perceptron (Simple MLP), an ElemNet-like model and an ElemNet-based transfer-learning model pretrained on the Matbench Materials Project formation-energy dataset, were further evaluated as supplementary benchmarks (Fig. S3 and Table S2). Comparison of the R^2^, MAE and RMSE values further supported the selection of XGBoost for compositional screening. The superior performance of XGBoost can be attributed to its suitability for the present small-scale, low-dimensional, composition-only screening task. Its tree-based ensemble structure can efficiently capture non-linear and coupled anion effects in tabular data while reducing the risk of overfitting, while deep-learning methods remain valuable alternatives for larger and more chemically diverse datasets [44,45].

The XGBoost model predicted Li_40_F_4_N_2_O_4_P_6_S_2_ as the optimal interphase composition (Fig. 1d), with a formation energy of −8.1 eV, closely matching the DFT-calculated value of −7.8 eV. The configurational entropy of the anion sublattice was calculated using Δ*S*_config_ = −*R*  $\mathop \sum \nolimits_{i = 1}^n {x}_i\ ln\ {x}_i$, where *x*_i_ represents the molar fraction of each anion. The calculated Δ*S*_conf_​ of Li_40_F_4_N_2_O_4_P_6_S_2_ is 1.523 *R*, exceeding the commonly used high-entropy threshold of 1.5 *R*. The XGBoost model was further optimized through hyperparameter tuning with early stopping to ensure robust convergence and avoid overfitting (Fig. S4). The narrow, zero-centered residual distributions across all datasets (Fig. S5) confirm the reliability of the XGBoost model and the absence of systematic bias. The optimized XGBoost model was then applied to an expanded virtual composition library of 500 candidate Li-F-N-O-P-S interphase compositions (Fig. 1e). These candidates were ranked by predicted formation energies and compared with the original DFT dataset. None showed a lower predicted formation energy than Li_40_F_4_N_2_O_4_P_6_S_2_, supporting its selection as the most stable composition identified in this screening workflow. This virtual screening step enables efficient exploration of a larger compositional space that would otherwise require extensive first-principles calculations, highlighting the value of data-efficient materials design under limited-data conditions. To improve model interpretability, SHapley Additive exPlanations (SHAP) analysis was performed for the XGBoost model (Fig. S6). The SHAP summary plot shows the contribution direction and magnitude of each element to the predicted formation energy, while SHAP interaction analysis identifies pairwise compositional interactions among different anions. These results indicate that the XGBoost predictions are influenced by both individual anion fractions and pairwise compositional interactions. Feature importance analysis (Fig. 1f) reflects the contributions of F, N, O, P and S to interphase stability, highlighting their distinct atomic effects [27]. Overall, this compositional screening workflow demonstrates that a limited but carefully curated DFT dataset can support reliable prediction and guide the discovery of stable HEIs, provided that the model scope is clearly defined and the predictions are rigorously validated [40–42,44].

### Characteristics of the high-entropy interphase

To evaluate the effect of the high-entropy Li_40_F_4_N_2_O_4_P_6_S_2_ phase on the Li^+^ transport kinetics within the SEI, we performed calculations to compare the energy barriers of Li^+^ diffusion across the Li_40_F_4_N_2_O_4_P_6_S_2_ and several interphases containing single anions, including LiF, Li_3_N, Li_2_O, Li_3_P and Li_2_S (Fig. 2a). Calculations were conducted on the most stable interface of each crystal, where the adsorption of Li adatoms on the surface was analyzed to define the initial and final states of the diffusion path (Fig. S7). The Li^+^ migration barrier in the high-entropy Li_40_F_4_N_2_O_4_P_6_S_2_ is 0.11 eV, comparable to the 0.12 eV of Li_3_N and 0.16 eV of Li_3_P, which are fast ionic conductors with ionic conductivities of 10^−4^–10^−3^ S cm^−1^ at 25°C [46,47]. This value is markedly lower than that of the LiF phase with 0.43 eV, which is almost inert to Li^+^ transport [27,28]. To assess interfacial stability, DFT calculations were performed for lithium metal in contact with Li_40_F_4_N_2_O_4_P_6_S_2_ and the other interphases, and the interface energies are shown in Fig. 2b. The high-entropy Li_40_F_4_N_2_O_4_P_6_S_2_ interphase shows the lowest interface energy (−0.79 J m^−2^) with lithium metal, comparable to that of LiF (−0.75 J m^−2^) with high lithiophobicity [48], and significantly lower than those of Li_3_N (−0.49 J m^−2^) and Li_3_P (−0.52 J m^−2^) with lithiophilicity [46,49]. Further comparative calculations on interphases with progressively increasing configurational entropy (Fig. S8), including LiF, Li_40_F_34_N_2_, Li_40_F_26_N_2_O_4_, Li_40_F_8_N_2_O_4_P_6_ and Li_40_F_4_N_2_O_4_P_6_S_2_, show that higher configurational entropy is associated with a lower Li^+^ migration barrier and a more favorable interface energy between the interphase and lithium metal. These calculations indicate that the high-entropy Li_40_F_4_N_2_O_4_P_6_S_2_ phase acts as a fast-conducting component in the SEI, facilitating rapid Li^+^ transport, while its highly stable interface with lithium metal promotes uniform and reversible lithium plating/stripping.

**Figure 2.**
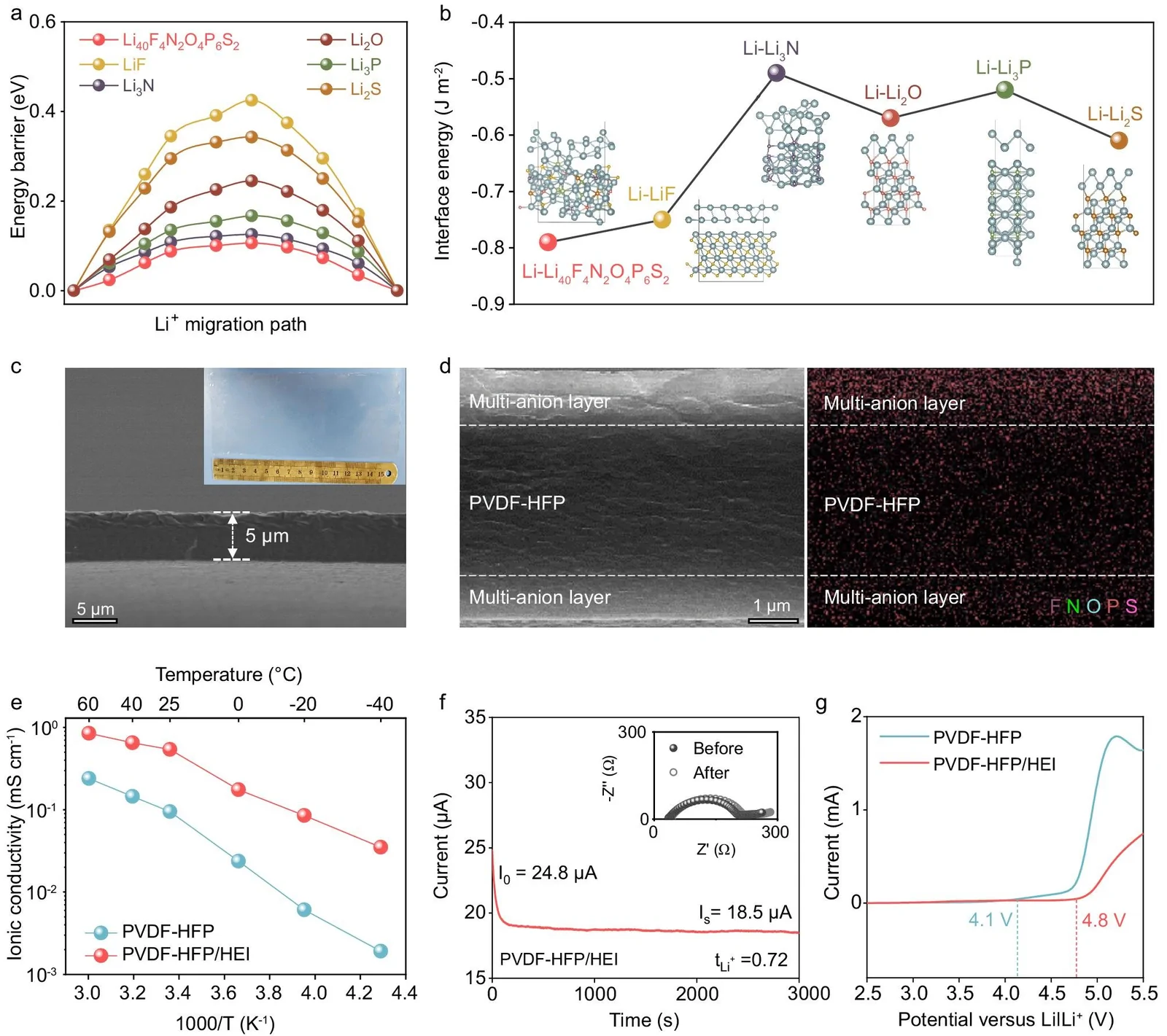
Characteristics of the HEI and the PVDF-HFP/HEI electrolyte. (a) Energy barriers of Li^+^ diffusion across different interphases. (b) Interface energies between lithium metal and different interphases. (c) Cross-sectional SEM image and digital photograph (inset) of the PVDF-HFP/HEI electrolyte. (d) Magnified cross-sectional SEM image and elemental maps of the PVDF-HFP/HEI electrolyte. (e) Arrhenius plots of ionic conductivity of the PVDF-HFP and PVDF-HFP/HEI electrolytes. (f) Current-time curve and Nyquist plots (inset) of the LiǀǀLi cell with the PVDF-HFP/HEI electrolyte. (g) Linear sweep voltammetry curves of the PVDF-HFP and PVDF-HFP/HEI electrolytes.

The anion ratios of the machine-learning-screened Li_40_F_4_N_2_O_4_P_6_S_2_ composition were used to quantitatively introduce F^−^, N^3−^, O^2−^, P^3−^ and S^2−^ multi-anion sources during electrolyte fabrication. PVDF-HFP/HEI, prepared via a multistep casting process (Fig. S9), forms a uniform, semi-transparent membrane with a thickness of 5 μm (Fig. 2c). Despite this ultrathin structure, it has superior mechanical properties, achieving a tensile strength of 8.9 MPa (Fig. S10), surpassing PVDF-HFP (3.1 MPa) and other reported thicker polymer electrolytes (Fig. S11). Magnified cross-sectional scanning electron microscopy (SEM) images and elemental maps (Fig. 2d and Fig. S12) show that the PVDF-HFP/HEI electrolyte consists of a PVDF-HFP substrate sandwiched between upper and lower multi-anion layers enriched with F, N, O, P and S elements. These multi-anion layers are designed to undergo interfacial reconstruction upon contact with lithium metal, thereby forming a chemically stable HEI in the SEI and enabling fast and uniform Li^+^ nucleation.

Surface SEM images show that the PVDF-HFP/HEI electrolyte has a dense and uniform morphology (Fig. S13), while the PVDF-HFP electrolyte is porous, indicating that multi-anion interactions enhance polymer chain adhesion and produce a compact structure that improves solvent uniformity and interfacial stability. Time of flight secondary ion mass spectrometry (TOF-SIMS) images (Fig. S14) further reveal homogeneous [Li(DMF)_x_]^+^ distribution in the PVDF-HFP/HEI electrolyte, while [Li(DMF)_x_]⁺ is aggregated in PVDF-HFP electrolyte. Thermogravimetric analysis shows [Li(DMF)_x_]⁺ contents of 5.7 wt% and 17.2 wt% in PVDF-HFP/HEI and PVDF-HFP, with the reduced [Li(DMF)_x_]^+^ residue helping to mitigate interfacial side reactions and enhance interface stability. The lower residual [Li(DMF)_x_]^+^ content in PVDF-HFP/HEI is mainly attributed to the improved membrane uniformity and denser morphology induced by the multi-anion sources, which suppress local accumulation of [Li(DMF)_x_]^+^ solvates. In addition, abundant anionic coordination sites weaken [Li(DMF)_x_]^+^ coordination and facilitate the removal of [Li(DMF)_x_]^+^-related species during vacuum drying.

Even with a lower residual content, the PVDF-HFP/HEI electrolyte has improved ionic conductivity across a wide temperature range from −40 to 60°C compared with the PVDF-HFP electrolyte (Fig. 2e and Fig. S15). Specifically, its ionic conductivity at 25°C reaches 5.4 × 10^−4^ S cm^−1^, markedly higher than the 0.98 × 10^−4^ S cm^−1^ of the pure PVDF-HFP electrolyte and those of PVDF-HFP-based electrolytes containing a single component, including LiF, Li_2_O, Li_2_S, Li_3_P and Li_3_N (Fig. S16). It also outperforms electrolytes with anion ratios of low-entropy and medium-entropy interphases, including Li_40_F_34_N_2_, Li_40_F_26_N_2_O_4_ and Li_40_F_8_N_2_O_4_P_6_. This improvement originates from the incorporation of HEI, which helps facilitate Li^+^ transport. The activation energy for Li^+^ migration decreases from 0.34 eV in PVDF-HFP electrolyte to 0.20 eV in PVDF-HFP/HEI electrolyte (Fig. S17), while the Li^+^ transference number (t_Li⁺_) increases from 0.31 to 0.72 (Fig. 2f and Fig. S18), indicating improved Li^+^ transport efficiency. To evaluate the interfacial Li^+^ charge-transfer kinetics between the electrolyte and lithium metal, Tafel analysis was performed (Fig. S19). The PVDF-HFP/HEI electrolyte shows a higher exchange current density of 0.18 mA cm^−2^ than the pure PVDF-HFP electrolyte (0.11 mA cm^−2^), indicating faster interfacial Li^+^ transport. Moreover, the electrochemical stability window extends to 4.8 V (Fig. 2g), compared with 4.1 V for PVDF-HFP electrolyte, implying improved compatibility with high-voltage positive electrodes. Voltage-dependent electrochemical impedance spectroscopy (EIS) measurements show that the PVDF-HFP electrolyte has a pronounced increase in interfacial impedance after high-voltage polarization (Fig. S20), especially at 4.8 V, whereas the PVDF-HFP/HEI electrolyte shows a much smaller impedance increase. X-ray photoelectron spectroscopy (XPS) analysis of the high-voltage-side surface after polarization to 4.8 V further reveals weaker oxidation/decomposition-related signals for the PVDF-HFP/HEI electrolyte than for the PVDF-HFP electrolyte (Fig. S21). These results indicate that the HEI suppresses high-voltage interfacial degradation, thereby contributing to the expanded electrochemical stability window from 4.1 V for PVDF-HFP to 4.8 V for PVDF-HFP/HEI.

### Characterization of the high-entropy interphase in the SEI

The multi-anion layers modulate electrochemical reactions at the Liǀelectrolyte interface, promoting *in situ* reconstruction into an HEI and thereby altering the SEI characteristics. To investigate the morphology and chemical composition of the SEI formed at the Li|PVDF-HFP/HEI and Li|PVDF-HFP interfaces, cryogenic transmission electron microscopy (cryo-TEM) experiments were conducted. As shown in Fig. 3a, in Li|electrolyte|Cu cells, after lithium plating at 0.5 mA cm^−2^ for 10 h, lithium metal with SEI was deposited on a TEM Cu grid. During this process, the cell with PVDF-HFP/HEI electrolyte maintains a low resistance with minimal variation, indicating the formation of a stable SEI with good ionic conduction. In contrast, the cell with PVDF-HFP electrolyte shows a significant increase in resistance, indicating excessive interphase formation with poor ionic conduction. The cryo-TEM image of the SEI in a Li|PVDF-HFP|Cu cell (Fig. 3b) reveals unevenly distributed nanoparticles embedded in an organic matrix formed *in situ* from electrolyte decomposition, including polymer fragments and lithium salt/solvent. High-resolution TEM (HRTEM) and fast Fourier transform (FFT) images (Fig. 3c) identify that these nanoparticles are LiF, Li_3_N, Li_2_O, Li_3_P and Li_2_S, with non-uniform sizes of 3–15 nm. This SEI with heterogeneous multicomponents, due to the differences in stability and ionic conductivity among its phases (Fig. 3d), tends to induce uneven and sluggish Li^+^ flux at the Li|PVDF-HFP interface, promoting dendrite growth and hindering fast Li^+^ transport under high current densities.

**Figure 3.**
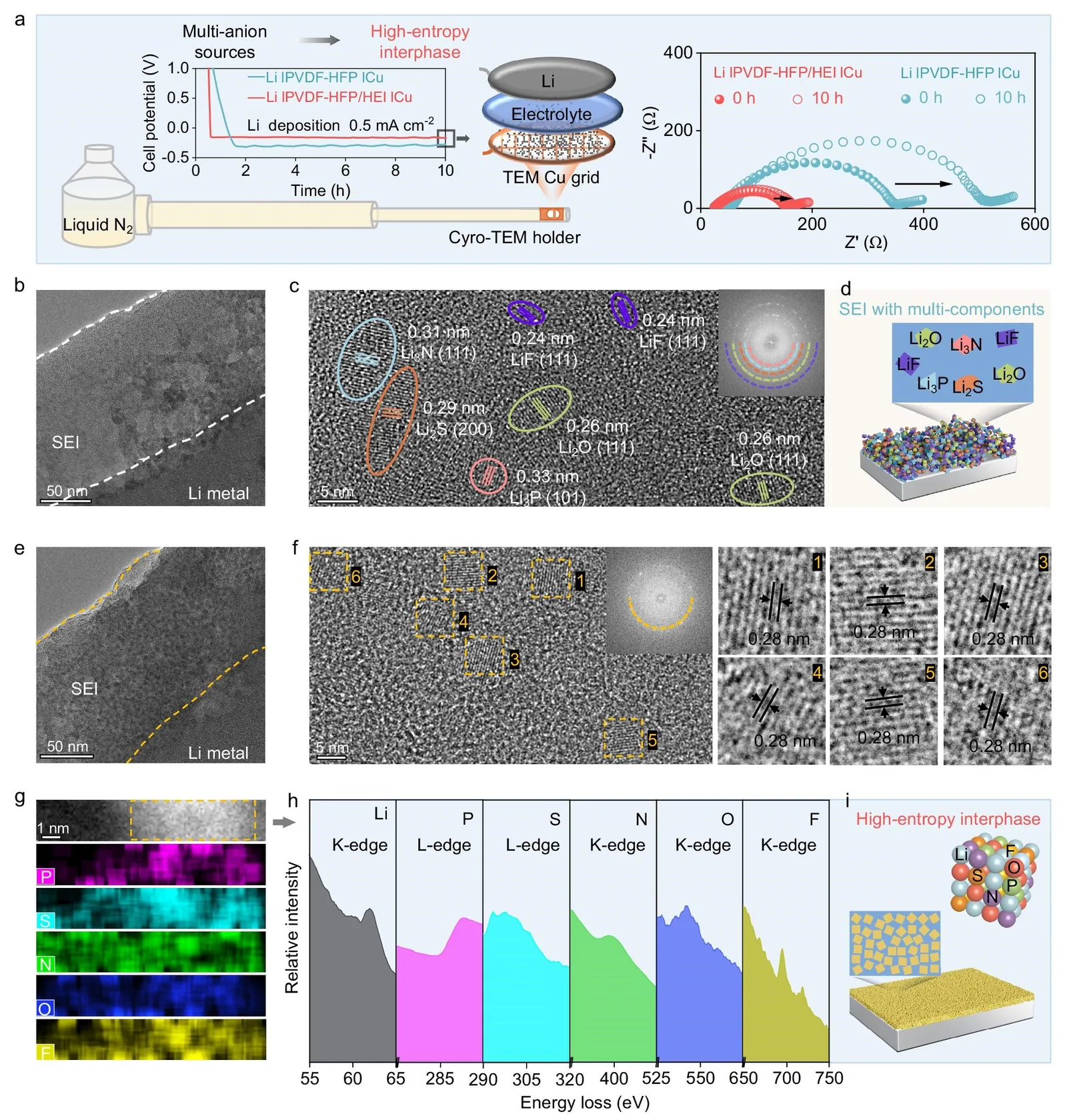
Cryo-TEM characterization of the SEI structure. (a) Schematic of the cryo-TEM experiment for characterizing SEI, along with the lithium deposition process of Li||Cu cells and the Nyquist plots before and after deposition. (b and c) TEM (b) and HRTEM (c) images of SEI formed when using PVDF-HFP electrolyte. (d) Schematic of SEI with multicomponents formed by PVDF-HFP electrolyte. (e and f) TEM (e) and HRTEM (f) images of SEI when using the PVDF-HFP/HEI electrolyte. (g and h) Elemental maps (g) and EELS spectra (h) of one inorganic nanoparticle in the SEI when using PVDF-HFP/HEI electrolyte. (i) Schematic of SEI with an HEI in the PVDF-HFP/HEI electrolyte.

When using the PVDF-HFP/HEI electrolyte, a dense SEI enriched with inorganic nanoparticles is formed, with the nanoparticles embedded in the organic matrix (Fig. 3e). These nanoparticles have remarkable uniformity, with an average size of 4–6 nm (Fig. S22). The HRTEM and FFT images (Fig. 3f) show that these nanoparticles possess a consistent lattice spacing of 0.28 nm, suggesting a homogeneous inorganic phase. The elemental maps (Fig. 3g) and electron energy-loss spectroscopy (EELS) analysis (Fig. 3h) of an individual inorganic nanoparticle further reveal its multi-anion composition. The elemental maps reveal the coexistence of P, S, N, O and F elements. Corresponding EELS spectra display distinct peaks from the Li K-edge, P L-edge, S L-edge, N K-edge, O K-edge and F K-edge, supporting the incorporation of lithium ions and multiple anions within the same nanoparticle (Fig. 3i). This uniform composition is enabled by the homogeneous multi-anion layers of the PVDF-HFP/HEI electrolyte, with an anion ratio designed according to the targeted Li-F-N-O-P-S composition of Li_40_F_4_N_2_O_4_P_6_S_2_. Upon contact with lithium metal and during lithium deposition, these surface-enriched anions participate in interfacial reconstruction, while entropy-stabilized mixing of F, N, O, P and S may help suppress phase separation and promote the formation of HEI-enriched nanoparticles within the SEI. Thermodynamically, multi-anion incorporation increases the configurational entropy of the interphase, thereby lowering the Gibbs free energy of mixing and stabilizing the high-entropy state. Consistently, the optimized Li_40_F_4_N_2_O_4_P_6_S_2_ composition exhibits a configurational entropy of 1.523 *R*, a low formation energy and a low interfacial energy with lithium metal, supporting the favorable thermodynamic and interfacial stability of the designed Li-F-N-O-P-S interphase. Moreover, after 100 cycles of lithium plating/stripping, the HEI nanoparticles remain uniformly distributed with similar sizes of 5–7 nm (Fig. S23), retain the characteristic lattice spacing of 0.28 nm, and still contain F, N, O, P and S within individual nanoparticles, indicating the structural and compositional stability of the HEI-enriched SEI during interfacial volume changes. These cryo-TEM results show that the synergistic incorporation of diverse anions induces the formation of a dense and uniform SEI enriched with a homogeneous multi-anion interphase with high-entropy characteristics, which imparts both fast interfacial ionic conduction and strong interfacial structural stability.

### Interface stability and electrochemical performance of SSLMBs

Using the PVDF-HFP/HEI electrolyte, symmetric LiǀǀLi cells sustain a high critical current density of 5.2 mA cm^−2^ (Fig. 4a), while the PVDF-HFP electrolyte fails as the current density is increased to 1.0 mA cm^−2^. As shown in Fig. 4b, a comparison of critical current densities with other thin electrolytes (see Table S3 for details) reveals that, despite being only 5 µm thick, the PVDF-HFP/HEI electrolyte delivers a higher critical current density than thicker counterparts. This is primarily attributed to the HEI, which stabilizes the Liǀelectrolyte interface and enables fast and uniform Li^+^ transport, while the uniformity and densification of the electrolyte further suppress lithium dendrite formation. In this process, structural stability and fast Li^+^ transport are coupled. The structurally stable HEI suppresses phase separation, thereby maintaining continuous Li^+^ transport pathways, while fast and homogeneous Li^+^ transport reduces concentration polarization and local current concentration, helping to preserve interfacial structural integrity during repeated lithium plating/stripping. These features support stable cycling performance at high current densities. With the PVDF-HFP/HEI electrolyte, symmetric LiǀǀLi cells achieve highly stable lithium stripping/plating over 6000 h at 0.5 mA cm^−2^ (Fig. 4c), maintaining a low and steady polarization below 100 mV. In contrast, the PVDF-HFP electrolyte, which has a lower ionic conductivity and limited interfacial stability, has continuously rising polarization and fails after 480 h. Moreover, PVDF-HFP electrolytes containing a single component, such as LiF, Li_2_O, Li_2_S, Li_3_P or Li_3_N, show unstable cycling behavior or large overpotentials in LiǀǀLi cells (Fig. S24), indicating that none of these components alone can simultaneously provide sufficient interfacial stability and fast Li^+^ transport. Similar instability or high polarization is also observed for PVDF-HFP electrolytes with anion ratio of low-entropy or medium-entropy interphases (Fig. S25). These comparisons highlight the importance of the HEI in stabilizing Li stripping/plating. To distinguish the high-entropy effect from simple multi-anion doping, we compared low-, medium- and high-entropy anion ratios containing the same F, N, O, P and S species. The high-entropy Li_40_F_4_N_2_O_4_P_6_S_2_ composition shows a lower Li^+^ migration barrier, more favorable lithium metal interface energy, more homogeneous SEI structure and more stable Li||Li cycling than the low- and medium-entropy counterparts (Fig. S26). These results indicate that the enhanced interfacial stability and Li^+^ transport originate from high configurational entropy rather than simple multi-anion doping. Accordingly, when the current density is increased to 1.0 mA cm^−2^ (Fig. S27) and 3.0 mA cm^−2^ (Fig. 4d), the PVDF-HFP/HEI electrolyte enables the LiǀǀLi cells to achieve stable cycling for 2000 and 850 h.

**Figure 4.**
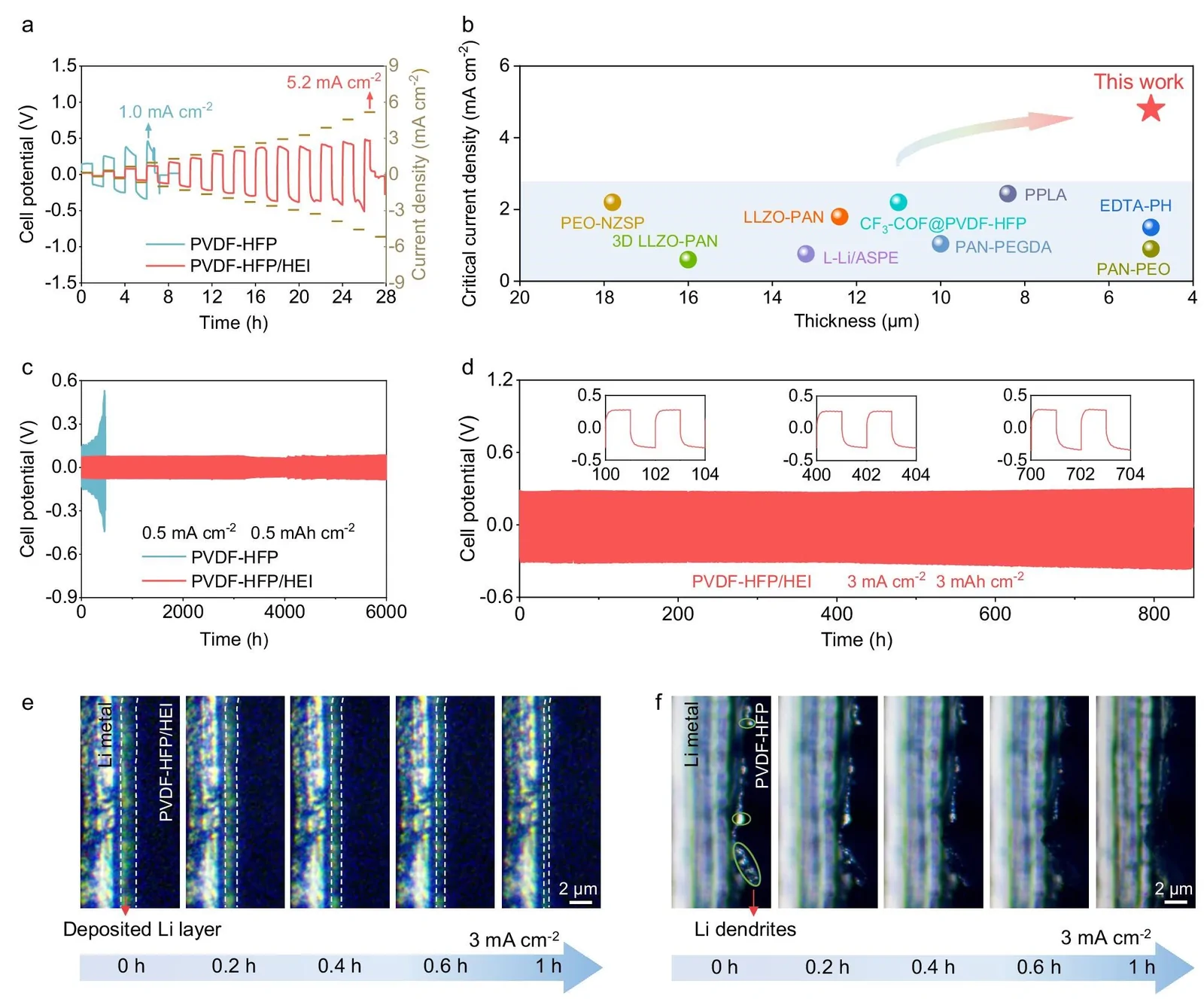
Electrochemical performance of the symmetric LiǀǀLi cells with the PVDF-HFP/HEI electrolyte at 25°C. (a) Critical current density of LiǀǀLi cells with PVDF-HFP and PVDF-HFP/HEI electrolytes. (b) Comparison of critical current densities of PVDF-HFP/HEI with other thin polymer-based electrolytes [8,16–18,50–54]. (c) Cycling performance of LiǀǀLi cells with PVDF-HFP and PVDF-HFP/HEI electrolytes at 0.5 mA cm^−2^. (d) Cycling performance of LiǀǀLi cells with PVDF-HFP/HEI electrolyte at 3 mA cm^−2^. (e and f) *In situ* optical microscopy images of LiǀǀLi cells with PVDF-HFP/HEI (e) and PVDF-HFP (f) electrolytes, observing lithium stripping at the Liǀelectrolyte interface in the first cycle at 3 mA cm^−2^.

To monitor and visualize in real time the interfacial evolution and stability of the Liǀelectrolyte interface under a practical current density, the lithium stripping process (Fig. S28) on the lithium metal side was observed by using *in situ* optical microscopy during the first cycle at 3 mA cm^−2^. From the recorded video (Video S1) and corresponding *in situ* images (Fig. 4e), the initially deposited lithium layer at the Li|PVDF-HFP/HEI interface has a smooth and uniform morphology. As the stripping process proceeds, this layer gradually becomes thinner, and no lithium dendrites are observed throughout the process. In contrast, the Li|PVDF-HFP interface initially fails to form a uniformly deposited lithium layer (Fig. 4f and Video S2), leading to pronounced lithium dendrites. The stripping process at 3 mA cm^−2^ over 1 h is also highly uneven, resulting in a rough and porous Li|PVDF-HFP interface after stripping. These *in situ* observations show that the PVDF-HFP/HEI electrolyte enables uniform and rapid Li^+^ flux even at high current densities, effectively suppressing the formation and growth of lithium dendrites. During the *in situ* experiments, the recorded Nyquist plots show that the LiǀǀLi cells with the PVDF-HFP/HEI electrolyte maintain a low and stable interfacial resistance (Fig. S29), whereas cells with PVDF-HFP electrolyte have higher resistance that increases markedly over time.

The PVDF-HFP/HEI electrolyte, with high interfacial stability and rapid Li^+^ transport capability, enables Li||LiNi_0.8_Co_0.1_Mn_0.1_O_2_ (Fig. 5) and Li||LiFePO_4_ (Figs S30–S33) solid-state batteries to achieve superior rate performance and long-term cycling stability at high current densities. Within the current density range of 0.05–5 mA cm^−2^, which corresponds to 0.1–10 C (Fig. 5a and b), Li||LiNi_0.8_Co_0.1_Mn_0.1_O_2_ cells with the PVDF-HFP/HEI electrolyte have higher capacities and minor voltage polarization than those using the PVDF-HFP electrolyte (Fig. S34). Specifically, the Li||LiNi_0.8_Co_0.1_Mn_0.1_O_2_ cells with the PVDF-HFP/HEI electrolyte show capacities of 202, 197, 172, 158, 144, 124, 108 and 84 mAh g^−1^ at 0.05, 0.1, 0.25, 0.5, 1, 2, 3 and 5 mA cm^−2^, respectively. When the current density is returned to 0.05 mA cm^−2^, the cells retain a high capacity of 200 mAh g^−1^.

**Figure 5.**
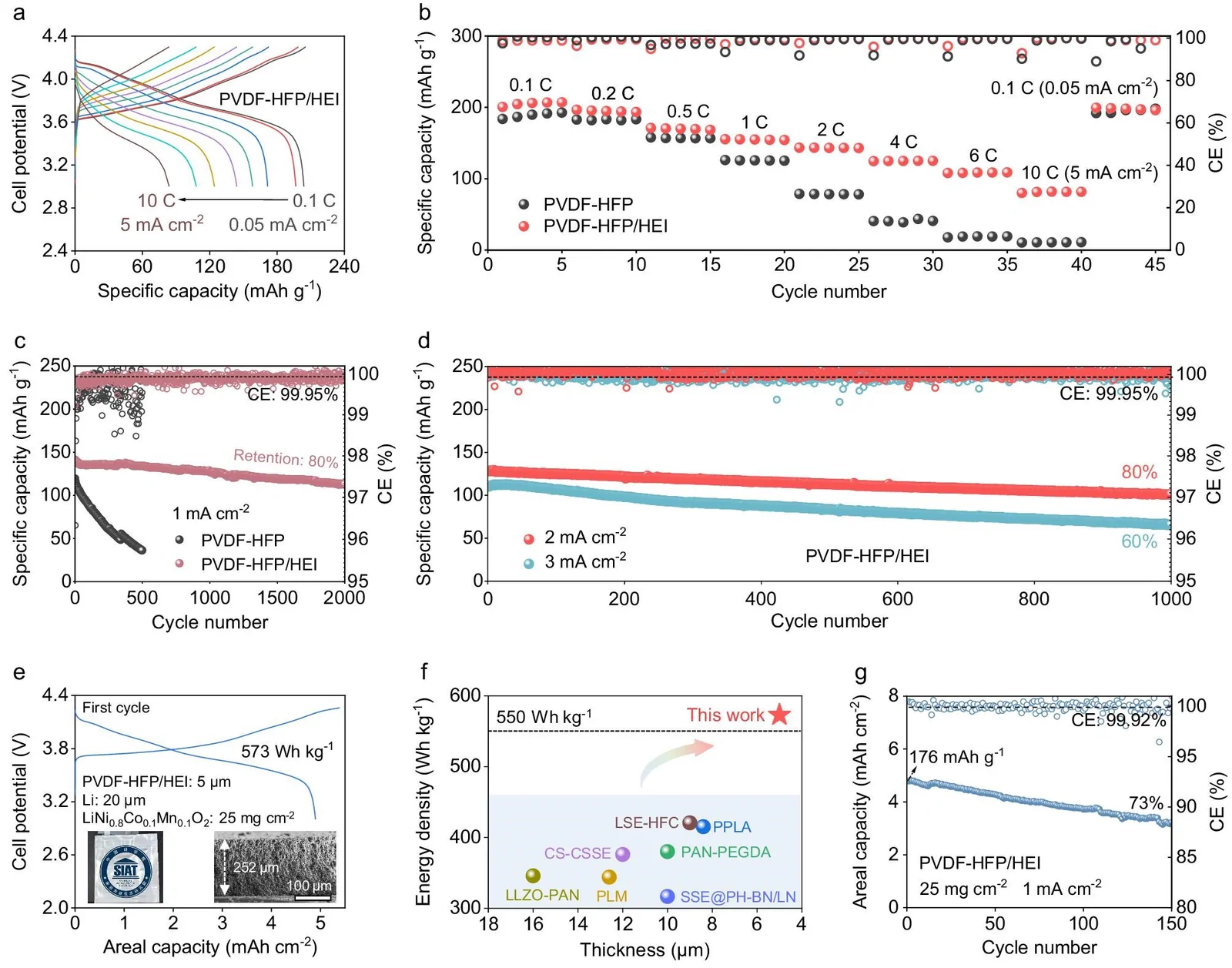
Properties of Li||LiNi_0.8_Co_0.1_Mn_0.1_O_2_ solid-state batteries, tested within 3.0–4.3 V at 25°C. (a) Charge/discharge profiles of cells with the PVDF-HFP/HEI electrolyte. (b) Rate capability of cells with the PVDF-HFP or PVDF-HFP/HEI electrolyte. (c and d) Long-term cycling performance of cells at 1 mA cm^−2^ with the PVDF-HFP or PVDF-HFP/HEI electrolyte (c) and at 2 and 3 mA cm^−2^ with the PVDF-HFP/HEI electrolyte (d). (e) Initial charge/discharge profile of a pouch cell with the PVDF-HFP/HEI electrolyte. (f) Comparison of the energy densities of solid-state batteries with the PVDF-HFP/HEI and other reported thin electrolytes [8,17,53,55–59]. (g) Cycling performance of a cell with a high areal capacity, using the PVDF-HFP/HEI electrolyte. The mass loading of LiNi_0.8_Co_0.1_Mn_0.1_O_2_ electrode is 2 mg cm^−2^ for panels (a–d), and 25 mg cm^−2^ for panels (e) and (g). The pouch cells in panels (e) and (g) were tested without external stacking pressure or clamping device during electrochemical performance evaluation.

In terms of cycling performance, Li||LiNi_0.8_Co_0.1_Mn_0.1_O_2_ cells with the PVDF-HFP/HEI electrolyte have much higher capacity retention and durability than those with the PVDF-HFP electrolyte (Fig. 5c and Fig. S35). At 1 mA cm^−2^, the PVDF-HFP/HEI-based cell shows an ultralong lifespan, retaining 80% of its initial capacity after 2000 cycles. The highly stable CEs of 99.95% indicate superior interfacial stability and minimal side reactions. In contrast, cells with the PVDF-HFP electrolyte suffer rapid capacity degradation and pronounced CE fluctuations, reflecting severe interfacial side reactions that are detrimental to cycling stability and Li^+^ transport efficiency. Morphological characterization of the PVDF-HFP/HEI electrolyte confirms its structural robustness with preserved integrity after 2000 cycles (Fig. S36), while the PVDF-HFP electrolyte experiences severe electrolyte structure damage after 500 cycles.

The cycling performance of the PVDF-HFP/HEI electrolyte was evaluated at higher current densities, as shown in Fig. 5d. At 2 mA cm^−2^, the Li||LiNi_0.8_Co_0.1_Mn_0.1_O_2_ cells have an 80% capacity retention after 1000 cycles. As the current density is increased to 3 mA cm^−2^, the initial capacity has a 60% capacity retention after 1000 cycles. In both cases, the CEs remain stable at 99.95%, further confirming the role of the uniform HEI in stabilizing the SEI and supporting efficient Li^+^ transport under high-current-density conditions. This stability is further supported by the preserved [Li(DMF)_x_]^+^ solvation environment in the PVDF-HFP/HEI electrolyte. After 1000 cycles at 3 mA cm^−2^, PVDF-HFP/HEI maintains a homogeneous [Li(DMF)_x_]^+^ distribution and nearly unchanged content of 5.7 wt% (Figs S14 and S37), whereas PVDF-HFP electrolyte shows aggravated aggregation and a decrease in [Li(DMF)_x_]^+^ content from 17.2 wt% to 11.3 wt%. These results indicate that the HEI suppresses [Li(DMF)_x_]^+^ decomposition and redistribution, thereby reducing interfacial side reactions during long-term cycling.

The ultrathin PVDF-HFP/HEI electrolyte minimizes the mass contribution of the electrolyte in the solid-state battery systems, thereby enhancing the overall energy density. At a thickness of 5 µm, its areal weight is only 0.58 mg cm^−2^, which is much lower than that of conventional polymer electrolytes (>30 µm, >3 mg cm^−2^) [60,61], which are typically required to suppress lithium dendrite growth under practical current densities. To meet the demand for high energy density in solid-state batteries, we assembled a pouch cell comprising the lightweight PVDF-HFP/HEI electrolyte, lithium foil with a thickness of 20 µm and thick LiNi_0.8_Co_0.1_Mn_0.1_O_2_ electrode with a high mass loading of 25 mg cm^−2^ (Fig. 5e). This configuration delivers an initial charge and discharge areal capacities of 5.4 and 4.9 mAh cm^−2^. Based on the total weight of the bare cell, including positive electrode, negative electrode, electrolyte and Al current collector (Table S4), an energy density of 573 Wh kg^−1^ is achieved, representing the highest values reported for solid-state batteries with thin electrolytes (Fig. 5f; see Table S5 for details). To assess practical safety, a fully charged Li||LiNi_0.8_Co_0.1_Mn_0.1_O_2_ pouch cell with the PVDF-HFP/HEI electrolyte was subjected to folding and cutting tests (Fig. S38). The pouch cell continuously powered a light-emitting diode during deformation and partial cutting, with no smoke, fire or explosion observed, showing good mechanical abuse tolerance and safety. Moreover, the Li||LiNi_0.8_Co_0.1_Mn_0.1_O_2_ cell with a high areal capacity exhibits stable cycling at 1 mA cm^−2^, retaining 73% of its initial capacity after 150 cycles with an average CE of 99.92% (Fig. 5g). To clarify the capacity decay mechanism under high mass loading, EIS measurements were conducted before and after cycling and compared with those of the cell with low mass loading. The cell with high mass loading shows a larger impedance after cycling, with a more pronounced increase in interphase/contact and charge-transfer resistances after cycling (Fig. S39), indicating that its capacity fading mainly arises from increased interfacial impedance, sluggish charge-transfer kinetics and Li^+^ transport limitations in the thick electrode. These results show that, although the HEI effectively stabilizes the Li|electrolyte interface and promotes uniform Li^+^ transport, practical high-areal-capacity cells still require further optimization of transport within thick electrodes.

## CONCLUSION

In summary, we develop a strategy to suppress dendrite penetration in ultrathin solid-state electrolytes under practical current densities by constructing an SEI with an HEI. Guided by machine learning screening, an optimized composition (Li-F-N-O-P-S) was incorporated into a 5-µm-thick PVDF-HFP/HEI electrolyte, forming an SEI enriched with a homogeneous interphase with high-entropy characteristics. This engineered HEI supports uniform and fast interfacial Li^+^ flux, enabling the ultrathin electrolyte to resist dendrite penetration at 5.2 mA cm^−2^ and maintain long-term cycling stability at a practical current density of 3 mA cm^−2^. Li||LiNi_0.8_Co_0.1_Mn_0.1_O_2_ cells show high cycling CEs of 99.95%, indicating superior interfacial stability and minimal side reactions. Pouch cells using this ultrathin electrolyte and a high-mass-loading LiNi_0.8_Co_0.1_Mn_0.1_O_2_ electrode deliver an energy density of 573 Wh kg^−1^. This work highlights that HEI engineering provides a dual-function benefit, reinforcing interfacial stability and promoting fast Li^+^ transport, offering an effective strategy for ultrathin electrolytes to meet the demanding requirements of practical current density.

## MATERIALS AND METHODS

### General information

Additional details on the materials, the characterization techniques employed and the electrochemical measurements are presented in the Supporting Information.

### Machine-learning-driven compositional ratios of the high-entropy interphase

We used an XGBoost-based machine learning framework with SHAP interpretation to screen the optimal composition of the HEI. The workflow included dataset generation, model benchmarking, hyperparameter tuning, final training, virtual screening and feature-importance analysis (see Materials and Methods in the online Supplementary file for details).

### DFT calculations

All DFT computations were carried out using the Vienna Ab initio Simulation Package. The Perdew-Burke-Ernzerhof functional within the generalized gradient approximation was employed to describe the exchange-correlation effects [62], and electron–ion interactions were treated using the projector-augmented wave method [63]. A plane-wave cutoff of 400 eV was applied throughout the calculations. The self-consistent field cycles were converged to 1 × 10^−5^ eV in total energy, with atomic forces optimized to below 0.03 eV Å^−1^. Dispersion interactions were incorporated using the Grimme D3 correction to improve the accuracy of structural optimizations [63].

Formation energies were calculated to guide the selection of anion types for the HEI and to compare the structural stability of HEIs with varying anion ratios. Li^+^ migration energy barriers across the HEI and other single-anion interphases were evaluated using the nudged elastic band method with eight intermediate images uniformly distributed along the diffusion pathway. Interface energies were calculated to assess the interfacial stability between the HEI and lithium metal, and were compared with those of other interphases in contact with lithium metal using (Eq. 1).


(1) \begin{equation*}
{E}_{{\mathrm{interface}}} = \frac{{\left[ {{E}_{{\mathrm{C}} + {\mathrm{D}}} - {\mathrm{\ }}{E}_{\mathrm{C}} - {\mathrm{\ }}{E}_{\mathrm{D}}} \right]}}{{2A}},
\end{equation*}

where *E*_C+D_, *E*_C_ and *E*_D_ denote the total energies of the combined surface and the two isolated surface slabs, respectively. *A* corresponds to the surface area used for the calculation.

### Preparation of PVDF-HFP and PVDF-HFP/HEI electrolytes

PVDF‑HFP and PVDF‑HFP/HEI electrolytes were prepared by solution casting and drying procedures. The PVDF-HFP/HEI electrolyte consisted of a PVDF‑HFP layer sandwiched between two multi‑anion layers with defined LiF, Li_3_N, Li_2_O, Li_3_P and Li_2_S ratios. Control electrolytes with varied anion compositions were fabricated identically (see Materials and Methods in the online Supplementary file for details).

### Materials characterization

The morphology and composition of the PVDF-HFP and PVDF-HFP/HEI electrolytes were examined using field‑emission SEM and TOF-SIMS. Mechanical properties were evaluated with a universal testing machine at room temperature, and thermal stability was assessed by thermogravimetric analysis. Surface chemical states were analyzed by XPS, with binding energy referenced to the C 1s peak. The SEI morphology and composition were characterized by cryo‑TEM equipped with EELS, using a liquid‑nitrogen holder and inert‑atmosphere transfer to prevent air exposure. Interfacial stability between lithium and electrolyte was monitored by *in situ* optical microscopy at 3 mA cm^−2^ (see Materials and Methods in the online Supplementary file for details).

## Supplementary Material

nwag511_Supplemental_Files

## References

[1] Janek J, Zeier WG. Challenges in speeding up solid-state battery development. Nat Energy 2023; 8: 230–40. 10.1038/s41560-023-01208-9

[2] Wan HL, Wang ZY, Zhang WR et al. Interface design for all-solid-state lithium batteries. Nature 2023; 623: 739–44. 10.1038/s41586-023-06653-w 37880366

[3] Wang J, Jia L, Du Y et al. Space charge regulation for ultra-stable all-solid-state lithium batteries by engineering of argyrodite electrolyte. Natl Sci Rev 2026; 13: nwag015. 10.1093/nsr/nwag015 PMC12988355 41835225

[4] Ding J, Zhang Y, Xu R et al. Review on lithium metal anodes towards high energy density batteries. Green Energy Environ 2023; 8: 1509–30. 10.1016/j.gee.2022.08.002

[5] Hong BL, Gao L, Li CP et al. All-solid-state batteries designed for operation under extreme cold conditions. Nat Commun 2025; 16: 143. 10.1038/s41467-024-55154-5 PMC11696891 39747842

[6] Li Z, Yu R, Weng ST et al. Tailoring polymer electrolyte ionic conductivity for production of low-temperature operating quasi-all-solid-state lithium metal batteries. Nat Commun 2023; 14: 482. 10.1038/s41467-023-35857-x PMC9886912 36717554

[7] Wang Q, Wang S, Lu TT et al. Ultrathin solid polymer electrolyte design for high-performance Li metal batteries: a perspective of synthetic chemistry. Adv Sci 2023; 10: 2205233. 10.1002/advs.202205233 PMC9811464 36442851

[8] Nie YH, Luo D, Yang TZ et al. Ultrathin electrolyte membranes with reinforced concrete structure for fast-charging solid-state lithium metal batteries. Adv Mater 2025; 37: 2504092. 10.1002/adma.202504092 40317846

[9] Wu JY, Yuan LX, Zhang WX et al. Reducing the thickness of solid-state electrolyte membranes for high-energy lithium batteries. Energy Environ Sci 2021; 14: 12–36. 10.1039/D0EE02241A

[10] Zhao Q, Cao ZJ, Wang XG et al. High-entropy laminates with high ion conductivities for high-power all-solid-state lithium metal batteries. J Am Chem Soc 2023; 145: 21242–52. 10.1021/jacs.3c04279 37751194

[11] Mi JS, Yang J, Chen LK et al. A ductile solid electrolyte interphase for solid-state batteries. Nature 2025; 647: 86–92. 10.1038/s41586-025-09675-8 41162700

[12] Yang S, Hu J, Jiang F et al. Safer solid-state lithium metal batteries: mechanisms and strategies. Infomat 2024; 6: e12512. 10.1002/inf2.12512

[13] Gao Y, Yan ZF, Gray JL et al. Polymer-inorganic solid-electrolyte interphase for stable lithium metal batteries under lean electrolyte conditions. Nat Mater 2019; 18: 384–9. 10.1038/s41563-019-0305-8 30858569

[14] Alsaç EP, Nelson DL, Yoon SG et al. Characterizing electrode materials and interfaces in solid-state batteries. Chem Rev 2025; 125: 2009–119. 10.1021/acs.chemrev.4c00584 PMC11869192 39903474

[15] Li H, Li M, Weng S et al. Autonomous ion-highways quasi-solid electrolytes toward high-voltage lithium metal batteries. Natl Sci Rev 2025; 12: nwaf363. 10.1093/nsr/nwaf363 PMC12485985 41040487

[16] Liu YH, Wang PH, Yang ZY et al. Lignin derived ultrathin all-solid polymer electrolytes with 3D single-ion nanofiber ionic bridge framework for high performance lithium batteries. Adv Mater 2024; 36: 2400970. 10.1002/adma.202400970 38623832

[17] Zhao XX, Wang C, Fan XM et al. Addressing the interface issues of all-solid-state lithium batteries by ultra-thin composite solid-state electrolyte combined with the integrated preparation technology. Infomat 2025; 7: e70012. 10.1002/inf2.70012

[18] Ma YX, Wan JY, Yang YF et al. Scalable, ultrathin, and high-temperature-resistant solid polymer electrolytes for energy-dense lithium metal batteries. Adv Energy Mater 2022; 12: 2103720. 10.1002/aenm.202103720

[19] Kim J, Kim Y, Ha Baek S et al. Two-dimensional polymeric metal phthalocyanines with anion fluxing and Li-ion-conducting properties for lithium metal full batteries. eScience 2026; 6: 100480. 10.1016/j.esci.2025.100480

[20] Liu Q, Zheng Z, Xiong P et al. Functional organic 7,7,8,8-tetracyanoquinodimethane artiffcial layers for the dendrite suppressed lithium metal anodes. Electron 2024; 2: e72. 10.1002/elt2.72

[21] Wang H, Yuan Y, Jia Q et al. A thin-layer, Li^+^ compensation, moisture-tolerant interfacial modification of Cu substrate toward the anode-less, energy/power dense Li metallic batteries. Adv Funct Mater 2024; 34: 2314186. 10.1002/adfm.202314186

[22] Shao A, Wang H, Zhang M et al. Multiscale interfacial stabilization via prelithiation separator engineering for ah-level anode-free lithium batteries. Nat Commun 2025; 16: 4145. 10.1038/s41467-025-59521-8 PMC12049431 40319029

[23] Zhang Q, Zhang X, Wan J et al. Homogeneous and mechanically stable solid-electrolyte interphase enabled by trioxane-modulated electrolytes for lithium metal batteries. Nat Energy 2023; 8: 725–35. 10.1038/s41560-023-01275-y

[24] Zhang Q, Li Y, Yu P et al. Accelerated defluorination kinetics for an ultrathin solid-electrolyte interphase in durable lithium metal batteries. J Am Chem Soc 2026; 148: 6915–25. 10.1021/jacs.5c16009 41418040

[25] Zeng K, Liu Q, Ma H et al. In situ co-growth LiF-Li_3_N rich dual-protective layers enable high interface stability for solid-state lithium-metal batteries. Energy Storage Mater 2024; 70: 103564. 10.1016/j.ensm.2024.103564

[26] Zheng J, Duan L, Ma H et al. Leveraging polymer architecture design with acylamino functionalization for electrolytes to enable highly durable lithium metal batteries. Energy Environ Sci 2024; 17: 6739–54. 10.1039/D4EE02218A

[27] Hobold GM, Wang CZ, Steinberg K et al. High lithium oxide prevalence in the lithium solid-electrolyte interphase for high Coulombic efficiency. Nat Energy 2024; 9: 580–91. 10.1038/s41560-024-01494-x

[28] Liu XS, Li SY, Yuan C et al. Probing the heterogeneous nature of LiF in solid-electrolyte interphases. Nature 2025; 646: 102–7. 10.1038/s41586-025-09498-7 40931064

[29] Dong C, Bi Z, Li R et al. Fluorine-doped argyrodite sulfide electrolyte enables commercial LiCoO_2_ use for 4.6 V high-voltage all-solid-state batteries. Natl Sci Rev 2025; 12: nwaf217. 10.1093/nsr/nwaf217 PMC12236154 40630822

[30] Tao F, Yan KJ, Dong CX et al. Electric-dipole coupling ion-dipole engineering induced rational solvation-desolvation behavior for constructing stable solid-state lithium metal batteries. Angew Chem Int Ed 2025; 64: e202503037. 10.1002/anie.202503037 40090904

[31] Xu JR, Li JM, Li YX et al. Long-life lithium-metal all-solid-state batteries and stable Li plating enabled by in situ formation of Li_3_PS_4_ in the SEI layer. Adv Mater 2022; 34: 2203281. 10.1002/adma.202203281 35765701

[32] Li YX, Song SB, Kim H et al. A lithium superionic conductor for millimeter-thick battery electrode. Science 2023; 381: 50–3. 10.1126/science.add7138 37410839

[33] Hull S, Keen DA, Sivia DS et al. Crystal structures and ionic conductivities of ternary derivatives of the silver and copper monohalides: I. Superionic phases of stoichiometry MA_4_I_5_:rbAg_4_I_5_, KAg_4_I_5_, and KCu_4_I_5_. J Solid State Chem 2002; 165: 363–71. 10.1006/jssc.2002.9552

[34] Li SH, Lin J, Schaller M et al. High-entropy lithium argyrodite solid electrolytes enabling stable all-solid-state batteries. Angew Chem Int Ed 2023; 62: e202314155. 10.1002/anie.202314155 37902614

[35] Bérardan D, Franger S, Meena AK et al. Room temperature lithium superionic conductivity in high entropy oxides. J Mater Chem A 2016; 4: 9536–41. 10.1039/C6TA03249D

[36] Dragoe N, Bérardan D. Order emerging from disorder. Science 2019; 366: 573–4. 10.1126/science.aaz1598 31672883

[37] Wang QS, Sarkar A, Wang D et al. Multi-anionic and -cationic compounds: new high entropy materials for advanced Li-ion batteries. Energy Environ Sci 2019; 12: 2433–42. 10.1039/C9EE00368A

[38] Ma PY, Kumar R, Wang KH et al. Active learning accelerates electrolyte solvent screening for anode-free lithium metal batteries. Nat Commun 2025; 16: 8396. 10.1038/s41467-025-63303-7 PMC12462480 40998777

[39] Wang BN, Doan HA, Son SB et al. Data-driven design of electrolyte additives supporting high-performance 5 V LiNi_0.5_Mn_1.5_O_4_ positive electrodes. Nat Commun 2025; 16: 3413. 10.1038/s41467-025-57961-w PMC11986164 40210879

[40] He B, Wang K, Jiang Z et al. Towards a sustainable high-quality materials data ecosystem: frameworks and strategies. Natl Sci Rev 2026; 13: nwag108. 10.1093/nsr/nwag108 PMC13105177 42040154

[41] Nandy A, Duan C, Kulik H. Audacity of huge: overcoming challenges of data scarcity and data quality for machine learning in computational materials discovery. Curr Opin Chem Eng 2022; 36: 100778. 10.1016/j.coche.2021.100778

[42] Liu Y, Yang Z, Zou X et al. A general framework to govern machine learning oriented materials data quality. Mater Sci Eng R Rep 2025; 166: 101050. 10.1016/j.mser.2025.101050

[43] He B, Jiang Z, Wang K et al. Multirole collaborative and co-constructive materials design ecosystem enabled by using control and data flows decoupled workflows. J Materiomics 2026; 12: 101177. 10.1016/j.jmat.2026.101177

[44] Liu Y, Yang Z, Zou X et al. Data quantity governance for machine learning in materials science. Natl Sci Rev 2023; 10: nwad125. 10.1093/nsr/nwad125 PMC10265966 37323811

[45] Saleem A, Shaw L, Chen Z et al. State-of-the-art machine learning technology for sustainable lithium battery cathode design: a perspective. Adv Energy Mater 2025; 15: 2405300. 10.1002/aenm.202405300

[46] Li WH, Li MS, Wang S et al. Superionic conducting vacancy-rich β-Li_3_N electrolyte for stable cycling of all-solid-state lithium metal batteries. Nat Nanotechnol 2025; 20: 265–75. 10.1038/s41565-024-01813-z PMC11835732 39587350

[47] Tu SB, Zhang B, Zhang Y et al. Fast-charging capability of graphite-based lithium-ion batteries enabled by Li_3_P-based crystalline solid-electrolyte interphase. Nat Energy 2023; 8: 1365–74. 10.1038/s41560-023-01387-5

[48] Zhang WR, Koverga V, Liu SF et al. Single-phase local-high-concentration solid polymer electrolytes for lithium-metal batteries. Nat Energy 2024; 9: 386–400. 10.1038/s41560-023-01443-0

[49] Bi ZJ, Sun QF, Jia MY et al. Molten salt driven conversion reaction enabling lithiophilic and air-stable garnet surface for solid-state lithium batteries. Adv Funct Mater 2022; 32: 2208751. 10.1002/adfm.202208751

[50] Ma SQ, Zhao N, Lin J et al. Flexible and ultra-thin membrane electrolyte with polymer-reinforced-ceramic ion-conducting framework for sodium metal batteries. J Energy Chem 2025; 104: 576–84. 10.1016/j.jechem.2025.01.015

[51] Yu FH, Mu YB, Han MS et al. Electrochemically stable and ultrathin polymer-based solid electrolytes for dendrite-free all-solid-state lithium-metal batteries. Mater Futures 2025; 4: 015101. 10.1088/2752-5724/ada0cc

[52] Feng G, Ma QY, Luo D et al. Designing cooperative ion transport pathway in ultra-thin solid-state electrolytes toward practical lithium metal batteries. Angew Chem Int Ed 2025; 64: e202413306. 10.1002/anie.202413306 39207276

[53] Zhang YN, Yu JM, Shi HS et al. Fiber-reinforced ultrathin solid polymer electrolyte for solid-state lithium-metal batteries. Adv Funct Mater 2025; 35: 2421054. 10.1002/adfm.202421054

[54] Liu LF, Shi YZ, Liu MY et al. An ultrathin solid electrolyte for high-energy lithium metal batteries. Adv Funct Mater 2024; 34: 2403154. 10.1002/adfm.202403154

[55] Zhang Z, Gou JR, Cui KX et al. 12.6 μm-thick asymmetric composite electrolyte with superior interfacial stability for solid-state lithium-metal batteries. Nano-Micro Lett 2024; 16: 181. 10.1007/s40820-024-01389-2 PMC11052750 38668771

[56] Bao CS, Zheng CJ, Wu MF et al. 12 μm-thick sintered garnet ceramic skeleton enabling high-energy-density solid-state lithium metal batteries. Adv Energy Mater 2023; 13: 2204028. 10.1002/aenm.202204028

[57] Guo SY, Su YF, Yan K et al. Robust and adhesive laminar solid electrolyte with homogenous and fast Li-ion conduction for high-performance all-solid-state lithium metal battery. Adv Sci 2024; 11: 2404307. 10.1002/advs.202404307 PMC11321704 38874109

[58] Xia SX, Zhang XF, Jiang ZY et al. Ultrathin polymer electrolyte with fast ion transport and stable interface for practical solid-state lithium metal batteries. Adv Mater 2025; 37: 2510376. 10.1002/adma.202510376 PMC12464653 40576499

[59] Liu KX, Xie Y, Ding T et al. High-strength ultrathin solid-state electrolyte with ion-rectifying honeycomb-like skeleton for lithium metal batteries free from external pressure. Adv Funct Mater 2025; 36: e11558. 10.1002/adfm.202511558

[60] Zhang M, Wang HL, Shao AH et al. Enabling 4.5 V solid polymer batteries through a 10 μm, crosslinked polyether electrolyte. Adv Energy Mater 2024; 14: 2303932. 10.1002/aenm.202303932

[61] Liu J, Bao ZN, Cui Y et al. Pathways for practical high-energy long-cycling lithium metal batteries. Nat Energy 2019; 4: 180–6. 10.1038/s41560-019-0338-x

[62] Perdew JP, Burke K, Ernzerhof M. Generalized gradient approximation made simple. Phys Rev Lett 1996; 77: 3865–8. 10.1103/PhysRevLett.77.3865 10062328

[63] Kresse G, Joubert D. From ultrasoft pseudopotentials to the projector augmented-wave method. Phys Rev B 1999; 59: 1758–75. 10.1103/PhysRevB.59.1758

