# Supplementary material for: A 5-µm-thick electrolyte with a high-entropy interphase for practical solid-state lithium metal batteries
Source: Natl Sci Rev. 2026 Aug 25;13(19):nwag511. doi: 10.1093/nsr/nwag511 (PMC13692793; doi:10.1093/nsr/nwag511)
Supplement: Supplementary file 1 [file nwag511_supplemental_files.zip › Supplementary data.pdf]

Supplementary Information

A 5 μm-thick electrolyte with a high-entropy interphase for practical solid-state lithium metal batteries

Xuexia Lan[1,2,3,†], Yi Zeng[1,†], Ye Chen[2,†], Zhengjie Chen[2], Zhen Li[1,4], Xinyuan Hu[5], Ziyong Li[4], Qiutan Liu[1], Renzong Hu[4], Jing Peng[1,2,6,*] and Hui-Ming Cheng[1,7,8,*]

[1]Shenzhen Key Laboratory of Energy Materials for Carbon Neutrality, Institute of Technology for Carbon Neutrality, Shenzhen Institutes of Advanced Technology, Chinese Academy of Sciences, Shenzhen 518055, China;

[2]Faculty of Materials Science and Energy Engineering, Shenzhen University of Advanced Technology, Shenzhen 518107, China;

[3]School of Materials Science and Engineering, Hefei University of Technology, Hefei 230009, China;

[4]School of Materials Science and Engineering, Guangdong Provincial Key Laboratory of Advanced Energy Storage Materials, South China University of Technology, Guangzhou 510640, China;

[5]Department of Chemical Engineering, University College London, London WC1E 7JE, UK;

[6]State Key Laboratory of Precision and Intelligent Chemistry, University of Science & Technology of China, Hefei 230026, China;

[7]SKKU Global Research Center, Sungkyunkwan University, Suwon 16419, Republic of Korea;

[8]Shenyang National Laboratory for Materials Science, Institute of Metal Research, Chinese Academy of Sciences, Shenyang 110016, China

***Corresponding authors.** E-mails: jing.peng@siat.ac.cn; hm.cheng@siat.ac.cn

[†]Equally contributed to this work.

MATERIALS AND METHODS

Machine learning-driven compositional ratios of the high-entropy interphase

To accelerate the discovery of the optimal high-entropy interphase, we employed a machine learning-assisted compositional screening framework. The workflow consists of the following steps:

Dataset generation for anion composition screening. Before model training, 200 candidate high-entropy interphase compositions with different elemental ratios were generated. The normalized elemental fractions of F, N, O, P, and S were used as the input features, while the formation energies were used as the target labels for supervised machine learning. The formation energy was employed as a descriptor of thermodynamic stability for screening candidate high-entropy interphase compositions. Based on the compositional ratios of the constituent anions, 100 compositions were assigned to the training and validation datasets, which were further divided at an 80:20 ratio for model development and hyperparameter optimization. The remaining 100 compositions were reserved as an independent test dataset for evaluating model generalization performance. The independent test dataset was not involved in model training, hyperparameter optimization, or model selection.

Model benchmarking and hyperparameter optimization. Six machine-learning models (mean predictor, linear regression, ridge regression, support vector regression, random forest, and XGBoost), were first benchmarked. Automatic hyperparameter optimization was performed for each model using the Optuna framework. A five-fold cross-validation on the training set was employed to evaluate each hyperparameter configuration, with the average negative mean squared error (MSE) as the optimization target. The tree-structured parzen estimator sampler was used to explore the hyperparameter space. After comparing R^2, MAE, cross-validation MAE, and RMSE, XGBoost was selected as the final model. Its performance was confirmed on the independent test dataset. Additional deep-learning baselines, including a Simple MLP, an ElemNet-like, and an ElemNet-transfer learning variant pretrained on the Matbench Materials Project formation-energy dataset, were evaluated as supplementary benchmarks. All deep-learning models were trained using the

AdamW optimizer with MSE loss. The initial learning rate was set to 1×10^{-3} for Simple MLP and ElemNet-like models, and reduced to 5×10^{-4} during fine-tuning of the transfer-learning model. A weight decay of 1×10^{-4} was applied to improve generalization. The batch size was set to 16 for training on the target dataset and 128 for pretraining on the Matbench MP formation-energy dataset. Early stopping with a patience of 50-60 epochs was employed based on validation loss to prevent overfitting. All models were trained for a maximum of 300 epochs unless convergence was reached earlier.

Final XGBoost training and performance evaluation. XGBoost was selected for dedicated training on the training set, with manual hyperparameter fine-tuning and early stopping on the validation set to prevent overfitting. During training, the RMSE for both the training and validation datasets was recorded for each boosting round to monitor the learning process. The final model was comprehensively evaluated using R^2, MAE, and RMSE, while five-fold cross-validation was performed to assess model robustness. The optimized XGBoost model was then used to predict formation energies across the compositional space and identify the optimal high-entropy interphase compositions.

Virtual composition screening. After model validation, the optimized XGBoost model was used to screen an expanded virtual composition library containing 500 candidate Li-F-N-O-P-S interphase compositions. These virtual candidates were generated by varying the relative fractions of F, N, O, P, and S within the same compositional space. The candidates were ranked according to their predicted formation energies and compared with the original DFT dataset to assess whether lower-energy compositions could be identified within the expanded compositional space. This workflow enabled efficient exploration of a larger compositional space beyond the original DFT dataset while reducing the number of required first-principles calculations.

Model interpretation using SHAP. SHAP analysis was used to interpret the optimized XGBoost model. SHAP values were calculated for the normalized elemental fractions of F, N, O, P, and S to evaluate their contributions to the predicted formation

energy. SHAP interaction values were further used to examine pairwise compositional effects among different anion fractions.

Preparation of PVDF-HFP and PVDF-HFP/HEI electrolytes

PVDF-HFP (Aladdin, Mw ~400000) and LiTFSI (Aladdin, 99.9%), were purchased and used without further purification. The PVDF-HFP electrolyte was prepared by dissolving 4.8 g PVDF-HFP and 2.4 g LiTFSI in 20 ml DMF (Aladdin, ≥99.8%) solvent and then stirring for 12 h at 40°C to obtain a homogeneous solution. The solution was then doctor-bladed onto a glass plate and dried in a vacuum oven at 100°C for 24 h. The PVDF-HFP/HEI electrolyte consists of two multi-anion layers sandwiching a PVDF-HFP layer. Its fabrication involved the following processes: 4.8 g PVDF-HFP and 2.4 g LiTFSI were dissolved in 20 mL DMF and stirred for 12 h at 40°C. LiF (Aladdin, ≥99.9%), Li_3N (Aladdin, ≥99.9%), Li_2O (Aladdin, ≥99.9%), Li_3P, and Li_2S (Aladdin, ≥99.5%) were then added to the above DMF-based solution to form a homogeneous high-concentration multi-anionic slurry, followed by stirring for another 12 h at 40°C. The molar ratio of LiF, Li_3N, Li_2O, Li_3P and Li_2S was 4:2:4:6:2, corresponding to weights of 0.104 g, 0.07 g, 0.12 g, 0.312 g, and 0.092 g, respectively. Li_3P was synthesized under an inert atmosphere by reacting phosphorus powder with molten lithium metal and subsequently ground into powder. The multi-anionic slurry was doctor-bladed onto the upper and lower surfaces of the PVDF-HFP polymer, followed by vacuum drying at 100°C for 24 h to obtain the PVDF-HFP/HEI electrolyte. The thicknesses of the PVDF-HFP and PVDF-HFP/HEI electrolytes were both controlled at 5 μm. For electrochemical tests, the electrolyte membranes were punched into disks with a diameter of 16 mm, corresponding to an area of 2.01 cm^2. The areal density was 0.528 mg cm^2 for the PVDF-HFP electrolyte and 0.580 mg cm^2 for the PVDF-HFP/HEI electrolyte. The areal loading of the multi-anion layer, determined from the mass difference of the PVDF-HFP membrane before and after double-side coating, was controlled at 0.026 mg cm^2 per side, corresponding to a total loading of 0.052 mg cm^2 for both sides.

For comparison, PVDF-HFP-based electrolytes containing a single component, including PVDF-HFP/LiF, PVDF-HFP/Li_3N, PVDF-HFP/Li_2O, PVDF-HFP/Li_3P, and

PVDF-HFP/Li_2S, were prepared. PVDF-HFP-based electrolytes with low-entropy and medium-entropy interphases were prepared by introducing the corresponding anion sources according to the anion ratios of $Li_{40}F_{34}N_2$, $Li_{40}F_{26}N_2O_4$, and $Li_{40}F_8N_2O_4P_6$ interphases. All control electrolytes were fabricated using the same casting and drying procedures as the PVDF-HFP/HEI electrolyte.

Materials characterization

The morphology and composition of the PVDF-HFP and PVDF-HFP/HEI electrolytes were examined using a field-emission SEM (TESCAN GAIA3, 2016). TOF-SIMS measurements were conducted on the same instrument using a pulsed 30 keV Ga^+ ion beam with a current of 1 nA. Mechanical properties were evaluated using a Universal Testing Machine 2203 at 25 °C to investigate the mechanical properties of the electrolytes. Thermogravimetric analysis was conducted using a TA Q50 instrument. XPS was performed using a PerkinElmer PHI 5000C XPS system, with the C 1s peak at 284.8 eV used as the binding energy reference. The morphology and composition of the SEI were characterized by cryo-TEM (ThermoFisher Themis Z) at 80 kV, equipped with EELS and elemental mapping, using a Gatan single-tilt liquid nitrogen holder for the frost-free sample transfer at cryogenic temperatures. Li||Cu cells were disassembled in an Ar-filled glovebox, and the TEM Cu grid with deposited lithium was removed using plastic tweezers to avoid mechanical damage. This TEM Cu grid was then transported from the glovebox to the cryo-TEM holder, using a sample holder with an inert atmosphere. During this process, the TEM Cu grid remained fully protected from air exposure. For the morphology characterization of the cycled electrolytes, the membranes were transferred from the glovebox to the instrument without an inert-atmosphere sample holder, with air exposure limited to less than 2 minutes. The interfacial stability between lithium metal and the electrolyte was monitored using in-situ electrochemical optical microscopy (DeltaPix, DPX M6000), conducted at a current density of 3 mA cm^{-2}.

Electrochemical measurements

EIS tests were conducted on a CHI 760E electrochemical workstation over a frequency range of 1 MHz to 0.01 Hz at an amplitude of 10 mV and 12 points per

decade. Electrolytes were sandwiched between two stainless steel spacers and assembled into 2025-type coin-cells. The ionic conductivity (σ) of the electrolytes was calculated using (Eq. 2).

$$\sigma = \frac{L}{S\,R} \qquad \text{(S1)}$$

where L is thickness of the electrolytes, S is the effective contact area between the electrolyte and the stainless steel, and R is the electrochemical impedance from the Nyquist plot. The EIS measurements were conducted on three independent samples at each temperature, and the σ values shown in the figures represent the average value.

The activation energy (Ea) was calculated from the Arrhenius Eq. 3.

$$\ln(T) = \ln\sigma_0 - \left(\frac{E_a}{RT}\right) \qquad \text{(S2)}$$

where σ_0 is the pre-exponential factor.

Exchange current density was evaluated using Li||Li symmetric cells assembled with PVDF-HFP or PVDF-HFP/HEI electrolyte, and the corresponding Tafel plots were obtained from the polarization curves. The exchange current density was determined by extrapolating the linear Tafel region to zero overpotential based on the Butler-Volmer relationship. Linear sweep voltammetry measurements were conducted with the electrolyte sandwiched between a lithium foil and a stainless-steel spacer in 2025-type coin cells, at 25°C. The Li^+ transference number was measured by direct-current polarization of symmetric Li||Li cells under a 10 mV voltage bias at 25°C, and calculated based on Eq. 4:

$$t_{Li^+} = \frac{I_S(\Delta V - I_0 R_0)}{I_0(\Delta V - I_S R_S)} \qquad \text{(S3)}$$

where I_0 and R_0 are the current and resistance of the symmetric Li||Li cells at initial state, I_s and R_s represent the current and resistance at steady state, ΔV is the applied voltage polarization applied.

The critical current density test of the electrolytes was conducted using symmetric Li||Li coin cells, with current density increased from 0.1 mA cm^{-2} to 10.0 mA cm^{-2} at 25°C on a Land battery testing system. Cycling stability of symmetric Li||Li coin cells were performed at 0.5, 1, and 3 mA cm^{-2}, corresponding to areal capacities of 0.5, 1,

and 3 mAh cm^{-2}, respectively, tested at 25°C on a Land battery testing system.

Fabrication of the solid-state lithium batteries

The $LiNi_{0.8}Co_{0.1}Mn_{0.1}O_2$ and $LiFePO_4$ positive electrodes were prepared by mixing active material $LiNi_{0.8}Co_{0.1}Mn_{0.1}O_2$ (Canrd, average particle size ~5 μm, ≥99% purity) or $LiFePO_4$ (Canrd, average particle size ~2 μm, ≥99% purity), Super P (Canrd, average particle size ~40 nm, ≥99% purity), polyvinylidene fluoride (Canrd, ≥99% purity), and LiTFSI (Aladdin, 99.9%) in a weight ratio of 90:3:3:4 in N-methyl-2-pyrrolidone. The resulting slurry was cast onto Al foil and dried at 100°C for 12 h, yielding positive electrodes with a mass loading of ~2.0 or ~25 mg cm^{-2}. With the PVDF-HFP and PVDF-HFP/HEI electrolytes, solid-state Li||$LiNi_{0.8}Co_{0.1}Mn_{0.1}O_2$ coin cells were assembled in an Ar-filled glovebox and tested from 0.05 mA cm^{-2} to 5 mA cm^{-2} for rate performance, and cycled at 1, 2, and 3 mA cm^{-2} for long-term cycling stability. Similarly, solid-state Li||$LiFePO_4$ cells were assembled and tested from 0.17 mA cm^{-2} to 4.08 mA cm^{-2} for rate performance, and cycled at 0.34, 1.36, and 2.72 mA cm^{-2} for long-term cycling stability. Pouch cells were assembled in an Ar-filled glovebox, with a lithium foil (20 μm), an ultrathin PVDF-HFP/HEI electrolyte and a $LiNi_{0.8}Co_{0.1}Mn_{0.1}O_2$ positive electrode with a high mass loading of 25 mg cm^{-2}. The pouch cells were tested without external stacking pressure or clamping during electrochemical evaluation.

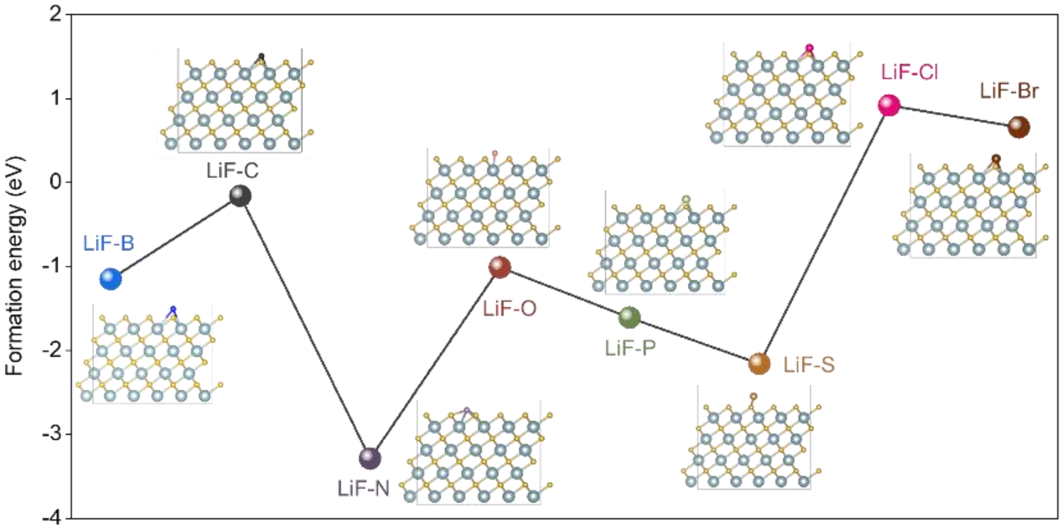

Figure S1. The formation energy of various anion-doped LiF structures.

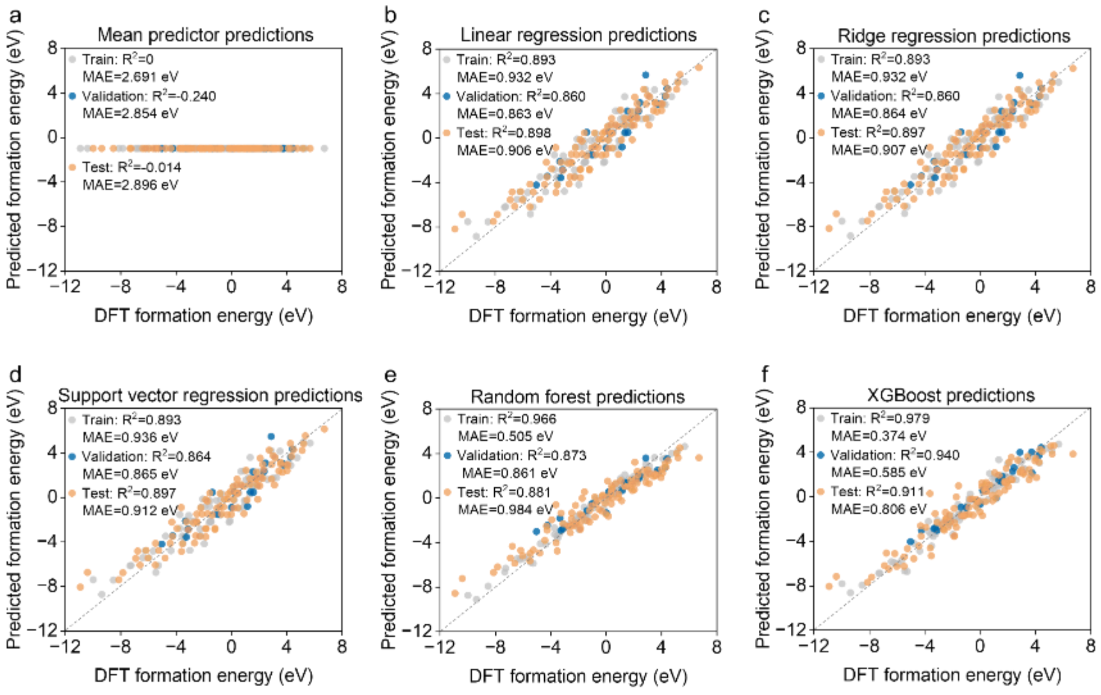

Figure S2. Predicted versus DFT-calculated formation energies for the benchmarked machine-learning models, including mean predictor (a), linear regression (b), ridge regression (c), support vector regression (SVR) (d), random forest (RF) (e), and XGBoost (f). Training, validation, and independent test samples are shown together for each model, with the grey dashed line indicating the ideal 1:1 linear relationship. All models were trained using the same composition-based feature set and optimized using the Optuna framework with five-fold cross-validation. XGBoost showed the best overall predictive performance and was therefore selected for subsequent compositional screening.

Table S1. Performance comparison for formation-energy prediction based on RMSE and five-fold CV MAE.

Model	Train RMSE (eV)	Validation RMSE (eV)	Test RMSE (eV)	CV MAE (eV)	CV MAE (eV/atom)
Mean predictor	3.362	3.224	3.579	2.780	0.0479
Linear regression	1.100	1.084	1.133	1.010	0.0174
Ridge regression	1.100	1.084	1.134	0.991	0.0171
Support vector regression	1.102	1.068	1.140	0.984	0.0170
Random forest	0.623	1.033	1.221	1.146	0.0198
XGBoost	0.491	0.712	1.054	0.982	0.0169

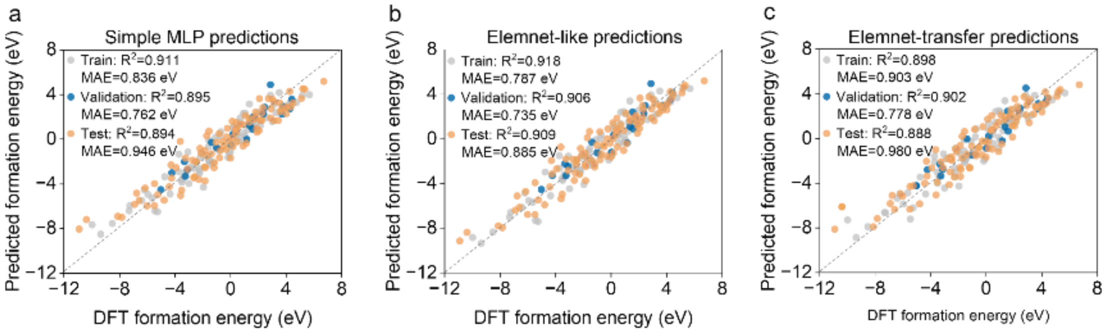

Figure S3. Predicted versus DFT-calculated formation energies for three deep-learning baseline models, including Simple MLP, ElemNet-like, and ElemNet-transfer learning models, with the grey dashed line indicating the ideal 1:1 linear relationship. Training, validation, and independent test samples are shown together for each model.

Table S2. The comparison of RMSE of Simple MLP, ElemNet-like, ElemNet-transfer and XGBoost models for formation-energy prediction.

Model	Train RMSE (eV)	Validation RMSE (eV)	Test RMSE (eV)
Simple MLP	1.003	0.937	1.159
ElemNet-like	0.965	0.887	1.072
ElemNet-transfer	1.076	0.905	1.191
XGBoost	0.491	0.712	1.054

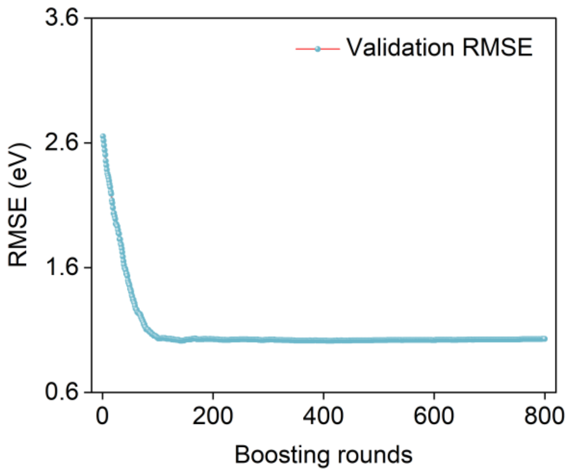

Figure S4. XGBboost learning curve: RMSE as a function of boosting rounds during model training. The curve demonstrates model convergence behavior and indicates whether additional training is needed or the model has reached optimal performance. Early stopping was employed to prevent overfitting by monitoring validation set performance.

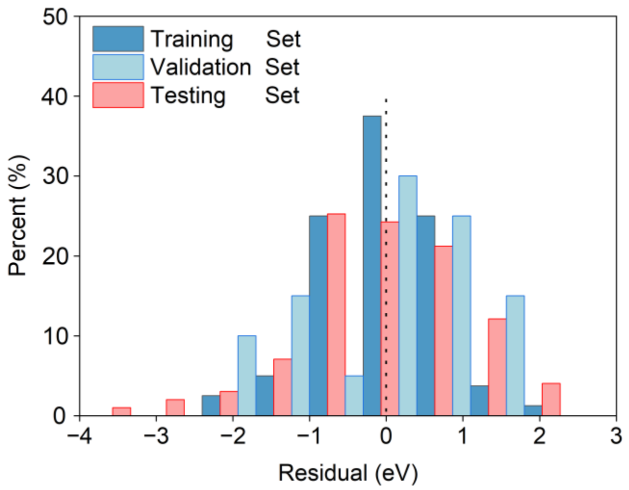

Figure S5. Residual distribution histograms for training, validation, and test sets. The vertical dashed line at zero indicates perfect prediction. The distribution shape reveals the model's prediction bias and error patterns across different data splits.

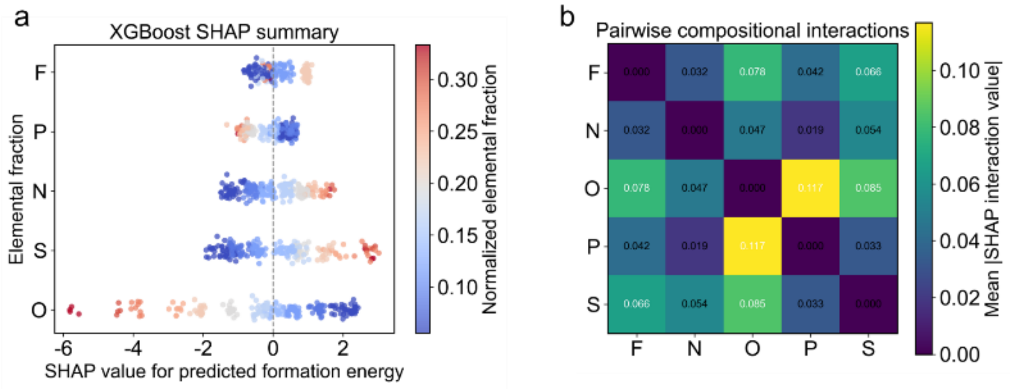

Figure S6. SHAP-based interpretation of the XGBoost model for high-entropy interphase formation-energy prediction. (a) SHAP summary plot showing the contribution of each elemental fraction to the predicted formation energy. Each point represents the SHAP value of one sample for a given elemental fraction, and the color denotes the corresponding normalized elemental fraction. Positive and negative SHAP values indicate contributions that increase and decrease the predicted formation energy, respectively. Oxygen shows the strongest overall influence on the model output, followed by sulfur, nitrogen, phosphorus, and fluorine. (b) Pairwise SHAP interaction matrix of elemental fractions. Each cell represents the mean absolute SHAP interaction value between two elements, with larger values indicating stronger coupled effects on the predicted formation energy. Diagonal entries are set to zero to highlight pairwise interactions. The analysis reflects model-captured compositional interactions rather than direct mechanistic causation.

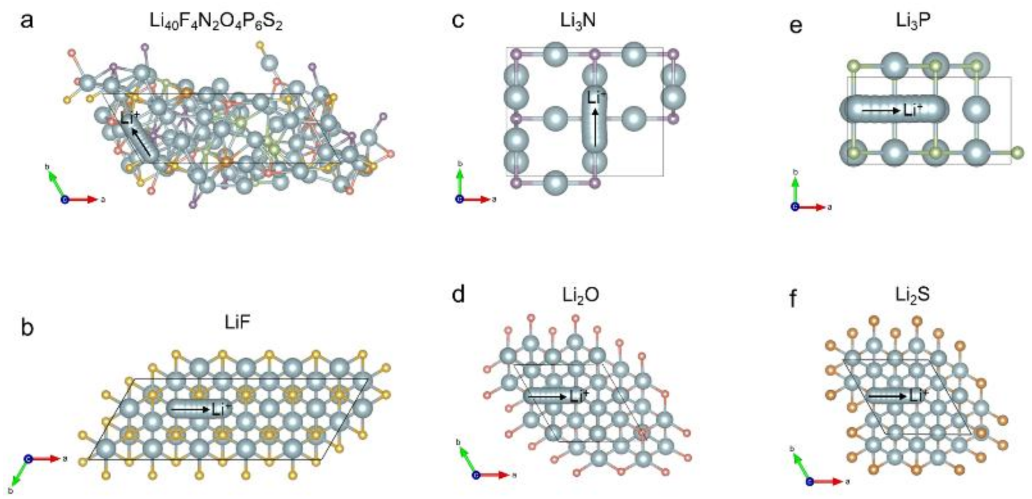

Figure S7. Diagram of the diffusion path of a Li^+ along (111) plane of $Li_{40}F_4N_2O_4P_6S_2$ (a), (111) plane of LiF (b), (110) plane of Li_3N (c), (111) plane of Li_2O (d), (100) plane of Li_3P (e), and (111) plane of Li_2S (f).

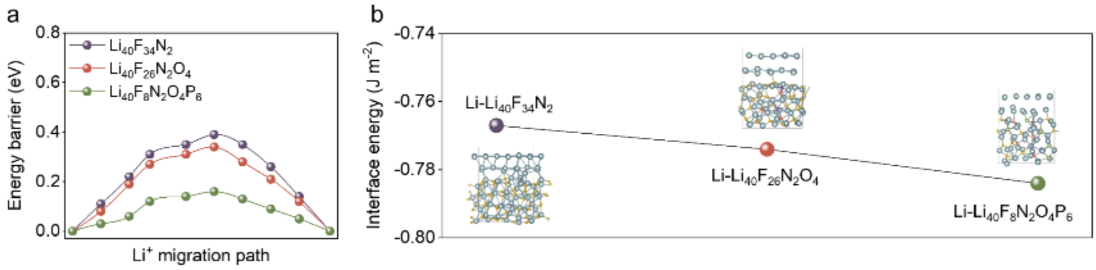

Figure S8. Li^+ diffusion barriers (a) and Li|interphase interface energies (b) of interphases with different configurational entropies. $Li_{40}F_{34}N_2$, $Li_{40}F_{26}N_2O_4$, and $Li_{40}F_8N_2O_4P_6$ have configurational entropies of 0.215 *R*, 0.602 *R*, and 1.280 *R*, respectively.

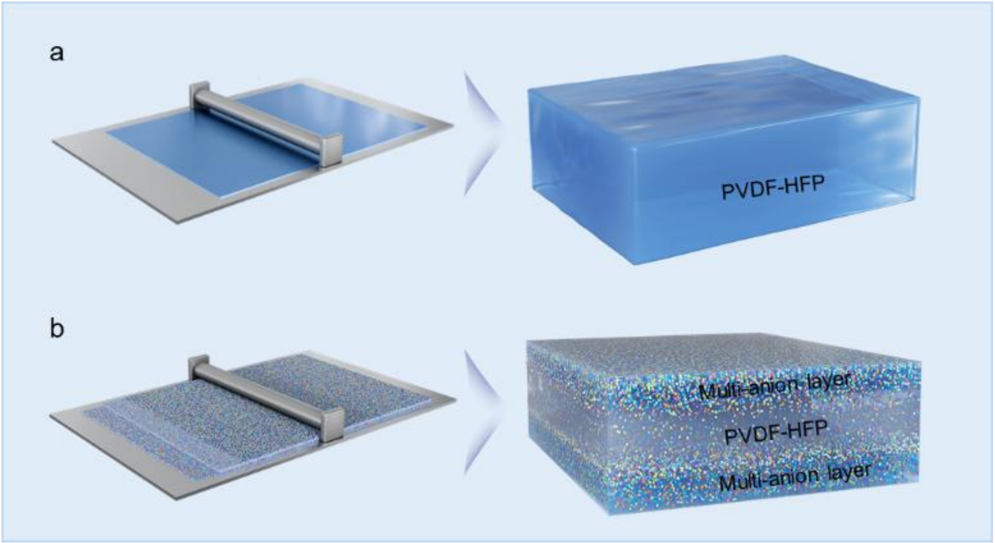

Figure S9. Fabrication process of PVDF-HFP (a) and PVDF-HFP/HEI (b) electrolytes.

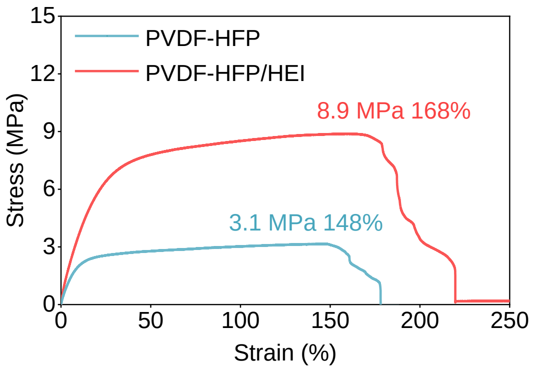

Figure S10. Stress-strain curves of PVDF-HFP and PVDF-HFP/HEI electrolytes.

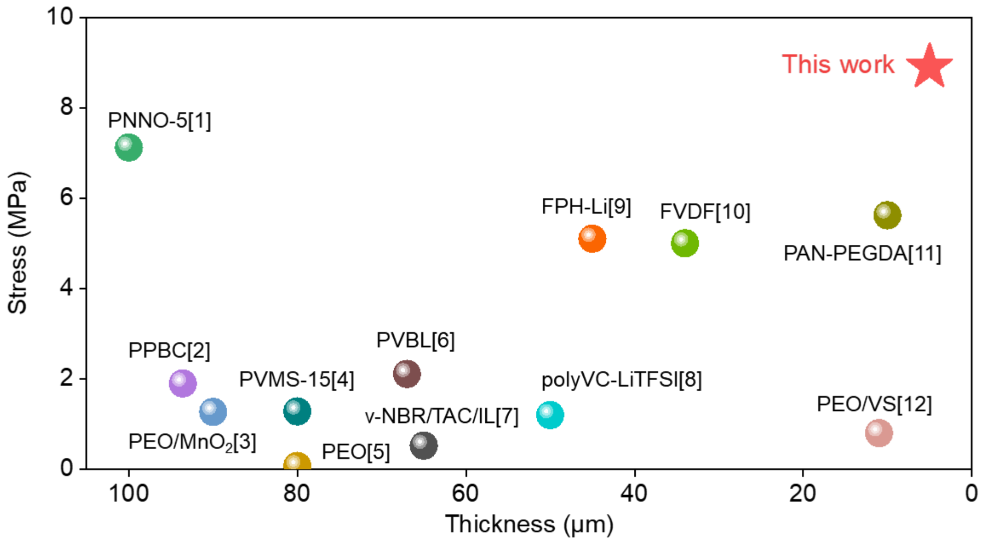

Figure S11. Comparison of the thickness and tensile strength of the PVDF-HFP/HEI and other polymer-based electrolytes[1-12]. PNNO-5, PPBC, PEO/MnO_2, PVMS-15, PVBL, v-NBR/TAC/IL, polyVC-LiTFSI, FPH-Li, PAN-PEGDA, PEO/VS stands for polyvinylidene fluoride (PVDF)/$NaNbO_3$, PVDF/poly(methyl methacrylate)/bacterial cellulose, polyethyleneoxide (PEO) with MnO_2 filer, PVDF with $MoSe_2$ filer, PVDF/$BaTiO_3$-$Li_{0.33}La_{0.56}TiO_{3-x}$, vulcanized nitrile butadiene rubber with triallyl cyanurate crosslinking agent and triallyl cyanurate an ionic liquid plasticizer, poly(vinylene carbonate) with LiTFSI salt, graphene-reinforced PVDF-HFP-LiTFSI, polyacrylonitrile fiber (PAN) with poly(ethylene glycol) diacrylate, and PEO with vermiculite sheets, respectively.

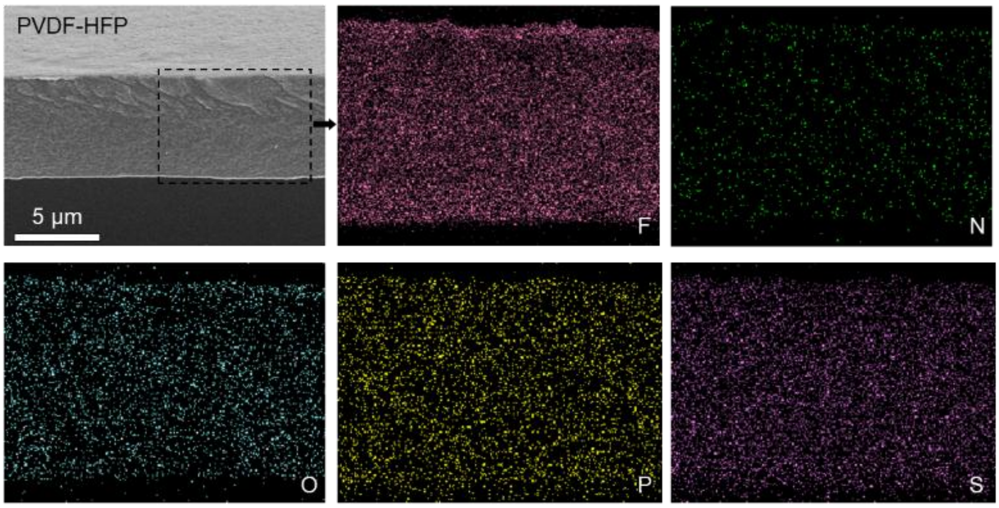

Figure S12. Cross-sectional SEM image and elemental maps of the PVDF-HFP electrolyte.

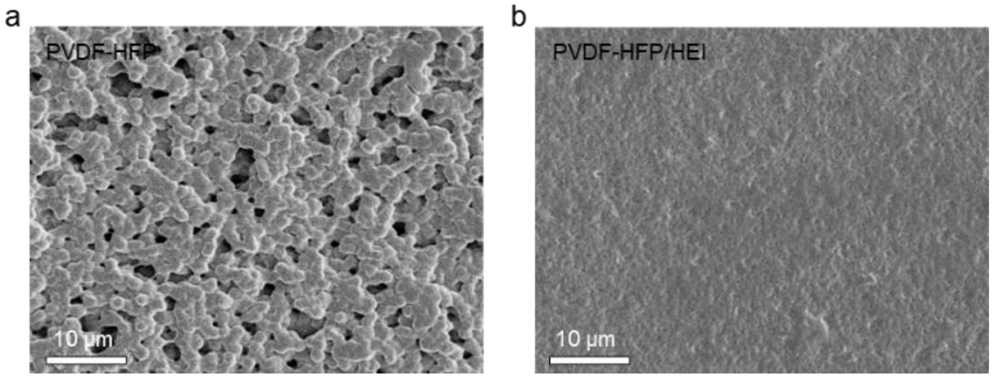

Figure S13. Surface SEM image of the PVDF-HFP (a) and PVDF-HFP/HEI (b) electrolytes.

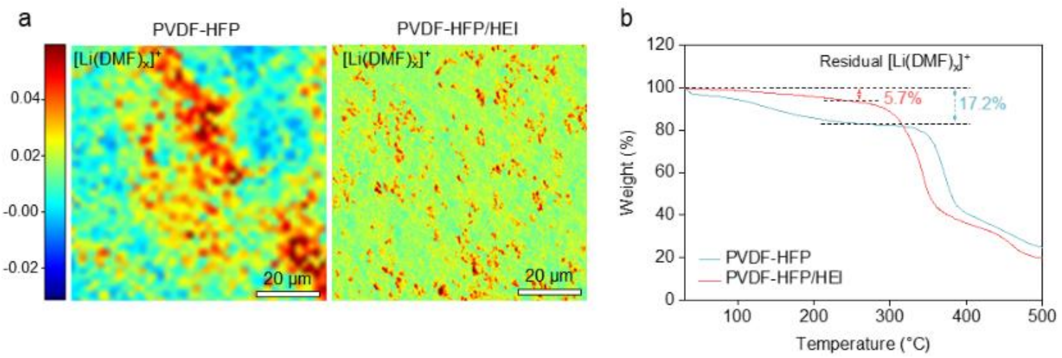

Figure S14. TOF-SIMS images showing the distribution of $[Li(DMF)_x]^+$ (a) and thermogravimetric analysis profiles (b) of PVDF-HFP and PVDF-HFP/HEI electrolytes. The first weight-loss stage in the thermogravimetric profiles is attributed to the evaporation of $[Li(DMF)_x]^+$, while the subsequent weight loss arises from the decomposition of the polymer and lithium salt.

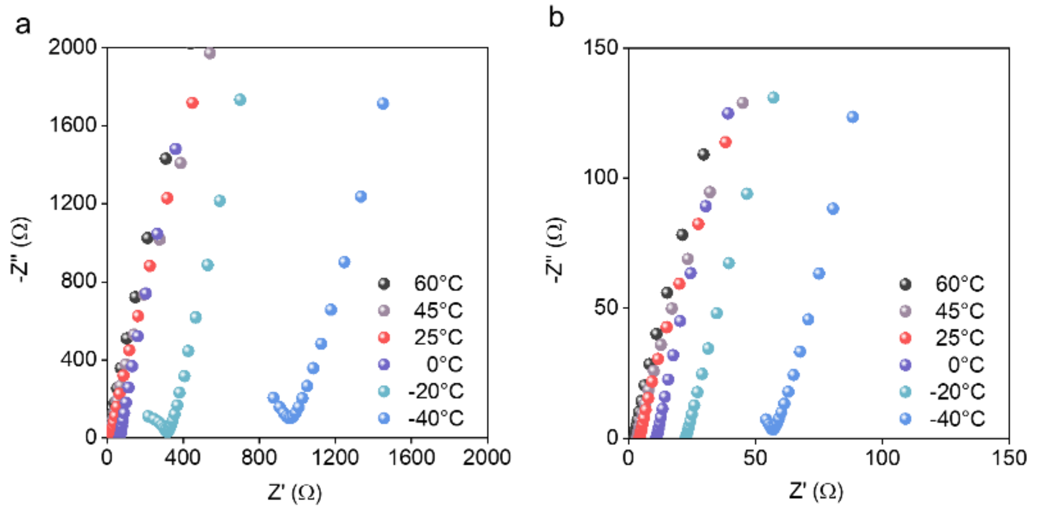

Figure S15. Nyquist plots measured for the PVDF-HFP (a) and PVDF-HFP/HEI (b) electrolytes at different temperatures.

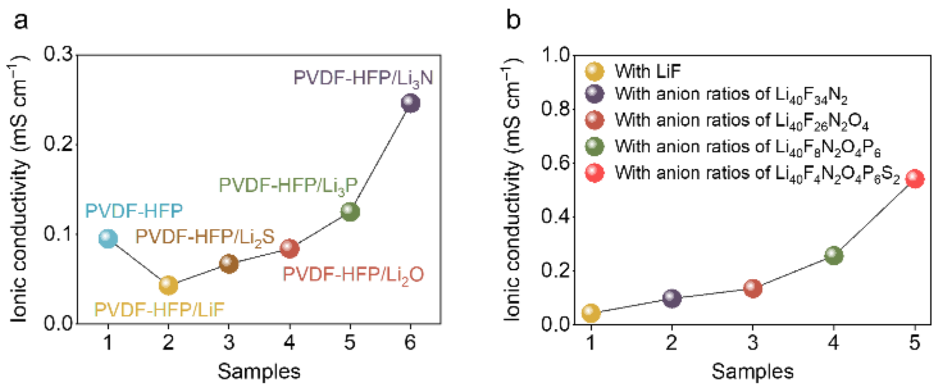

Figure S16. (a) Ionic conductivities of PVDF-HFP and PVDF-HFP electrolytes containing different single components. (b) Ionic conductivities of PVDF-HFP-based electrolytes. LiF, $Li_{40}F_{34}N_2$, $Li_{40}F_{26}N_2O_4$, $Li_{40}F_8N_2O_4P_6$, and $Li_{40}F_4N_2O_4P_6S_2$ have configurational entropies of 0 *R*, 0.215 *R*, 0.602 *R*, 1.280 *R*, and 1.523 *R*, respectively.

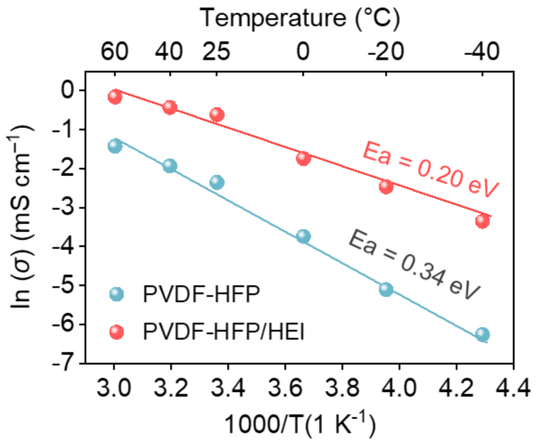

Figure S17. Arrhenius plots for the ionic conductivities of PVDF-HFP and PVDF-HFP/HEI electrolytes.

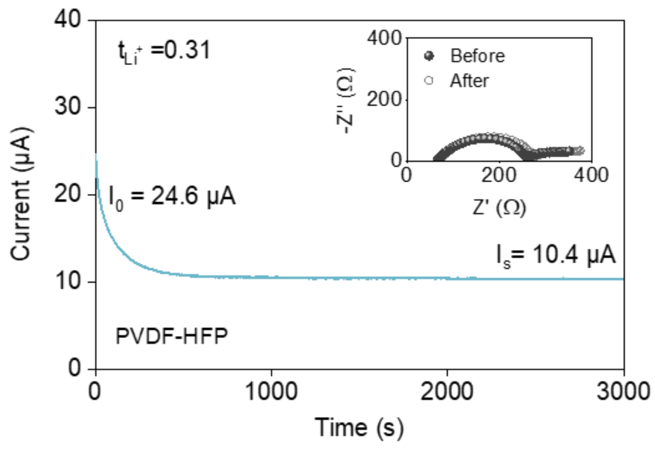

Figure S18. Current-time curves of the symmetric Li||Li cell with the PVDF-HFP electrolyte. The inset shows the Nyquist plots of the cell before and after polarization.

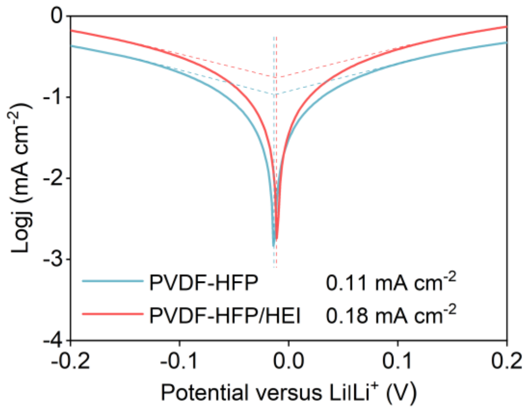

Figure S19. Tafel plots of cells using PVDF-HFP and PVDF-HFP/HEI electrolytes.

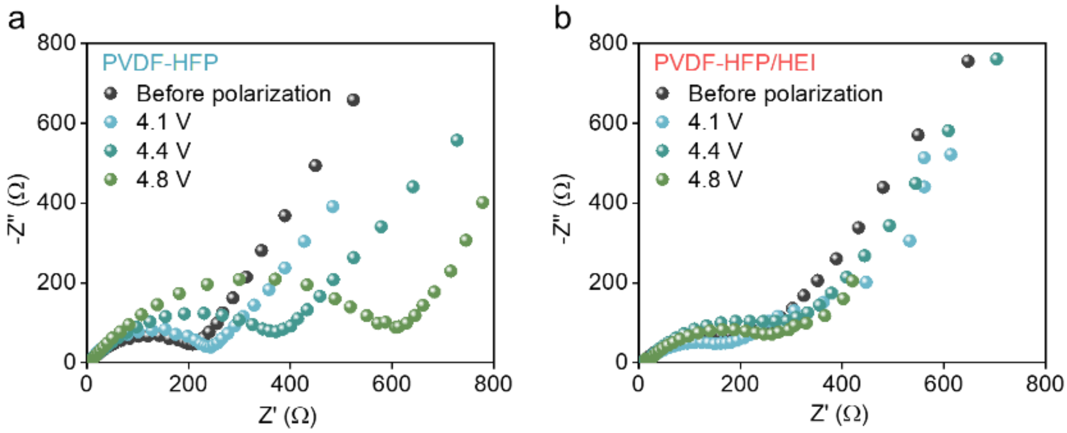

Figure S20. Voltage-dependent electrochemical impedance spectra of Li|electrolyte|SS cells in the linear sweep voltammetry test with PVDF-HFP (a) and PVDF-HFP/HEI (b) electrolyte before and after polarization at different voltages.

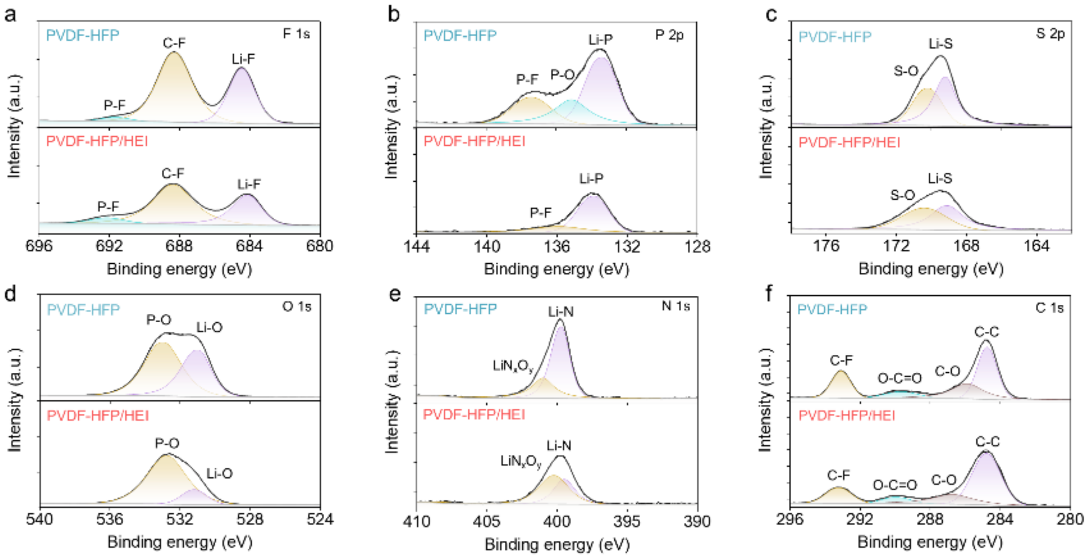

Figure S21. High-resolution XPS spectra of the high-voltage-side surfaces of PVDF-HFP and PVDF-HFP/HEI electrolytes after polarization to 4.8 V: (a) F 1s, (b) P 2p, (c) S 2p, (d) O 1s, (h) N 1s, and (f) C 1s spectra.

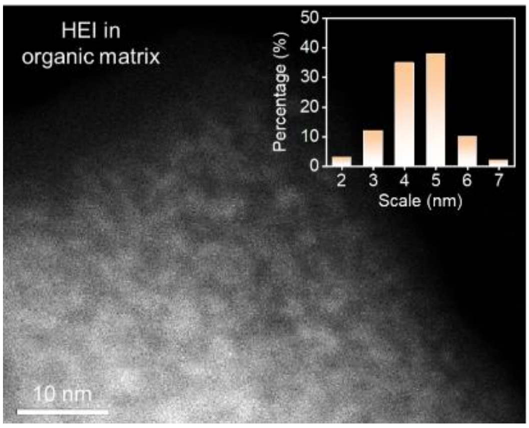

Figure S22. High angle annular dark field image of the SEI formed with the PVDF-HFP/HEI electrolyte. The inset shows the size distribution of the inorganic interphases.

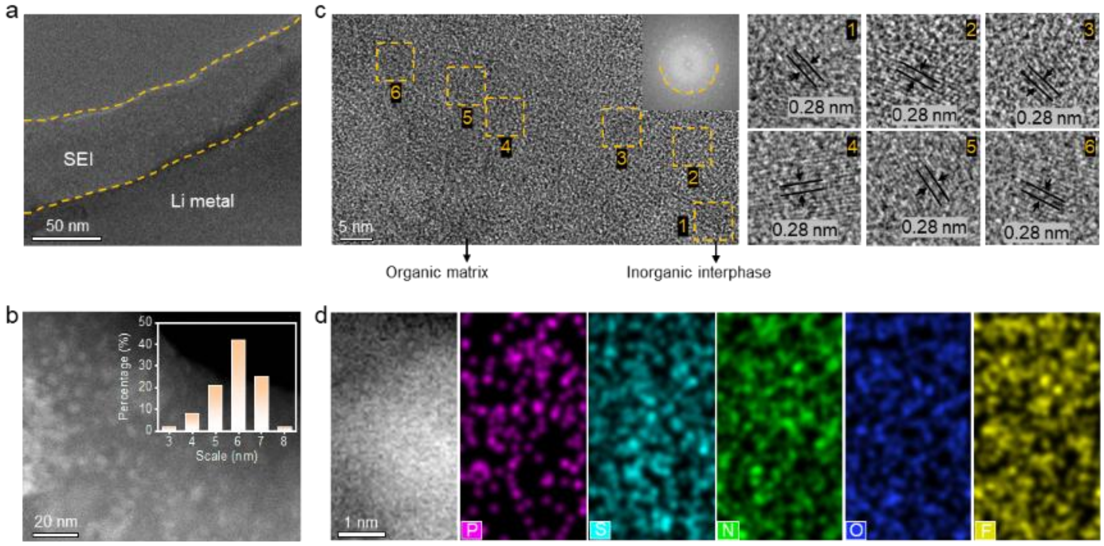

Figure S23. Cryo-TEM characterization of the SEI formed on the lithium metal anode after 100 cycles of repeated lithium plating/stripping with the PVDF-HFP/HEI electrolyte. (a) TEM image of the cycled SEI layer. (b) HAADF image of inorganic nanoparticles in the SEI. The inset shows the size distribution of the inorganic nanoparticles. (c) HRTEM image and FFT images of the inorganic nanoparticles in the SEI. (d) Elemental maps of an individual inorganic nanoparticle in the SEI.

Table S3. Comparison of critical current densities of PVDF-HFP/HEI with other thin polymer-based electrolytes.

Electrolyte	Thickness (μm)	Critical current density (mA cm^{-2})	Reference
PEO-$Na_3Zr_2Si_2PO_{12}$ (PEO-NZSP)	17.8	2.2	[13]
3D $Li_7La_3Zr_2O_{12}$-PAN (3D LLZO-PAN)	16	0.6	[14]
Lignin-based lithium salt-PVDF-HFP-PEO-LiTFSI (L-Li/ASPE)	13.2	0.76	[15]
$Li_{6.5}La_3Zr_{1.5}Ta_{0.5}O_{12}$-PAN (LLZO-PAN)	12.4	1.8	[16]
Fluorinated covalent organic framework@PVDF-HFP (CF_3-COF@PVDF-HFP)	11	2.2	[17]
PAN-PEGDA	10	1.04	[11]
PVDF-HFP/Pebax/LiTFSI/$Li_{6.25}Al_{0.25}La_3Zr_2O_{12}$ (PPLA)	8.4	2.44	[18]
PAN-PEO	5	0.9	[19]
EDTA-PH	5	1.5	[20]
This work (PVDF-HFP/HEI)	5	5.2	

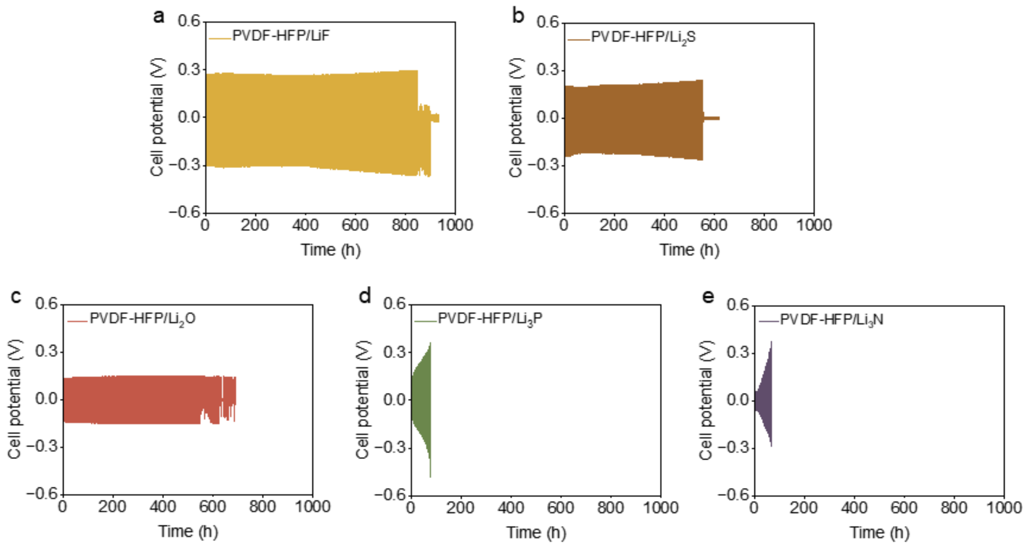

Figure S24. Cycling performance of Li||Li cells with PVDF-HFP electrolytes containing different single components, tested at 0.5 mA cm^{-2} and 25°C.

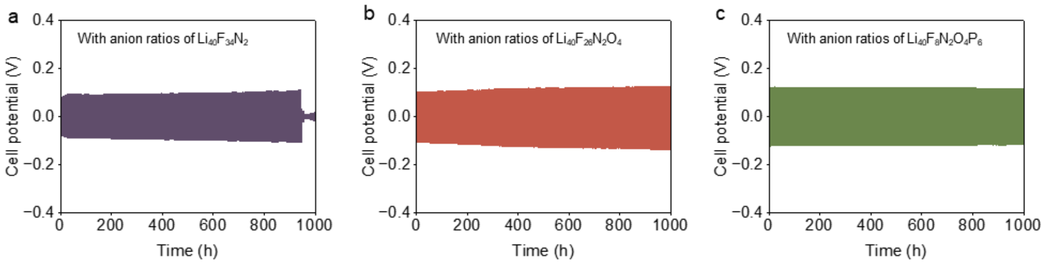

Figure S25. Cycling performance of Li||Li cells with PVDF-HFP-based electrolytes, tested at 0.5 mA cm^{-2} and 25°C. $Li_{40}F_{34}N_2$, $Li_{40}F_{26}N_2O_4$, and $Li_{40}F_8N_2O_4P_6$ have configurational entropies of 0.215 *R*, 0.602 *R*, and 1.280 *R*, respectively.

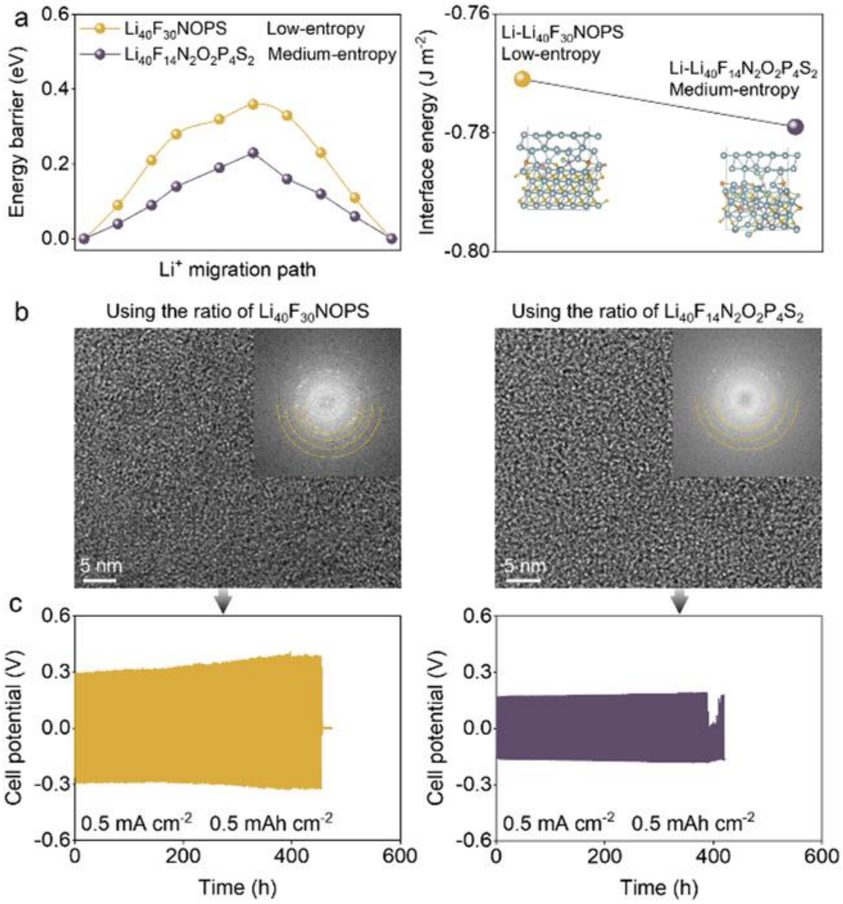

Figure S26. (a) DFT-calculated Li^+ migration energy barriers and interface energies with Li metal for $Li_{40}F_{30}NOPS$ and $Li_{40}F_{14}N_2O_2P_4S_2$, corresponding to low- and medium-entropy anion ratios, respectively. (b) HRTEM and corresponding FFT images of SEIs formed from electrolytes with low- and medium-entropy anion ratios. (c) Cycling performance of Li||Li cells using electrolytes prepared with low- and medium-entropy anion ratios.

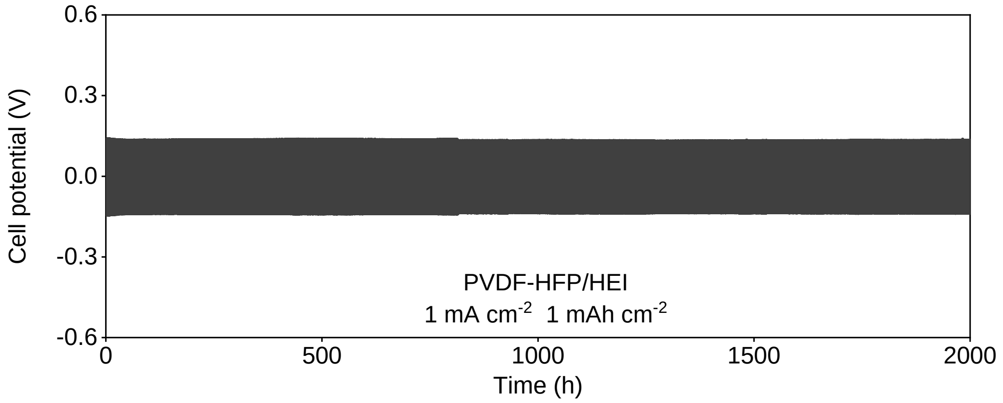

Figure S27. Long-term cycling performance of symmetric Li||Li cells with the PVDF-HFP/HEI electrolyte, tested at 1 mA cm^{-2} and 25°C.

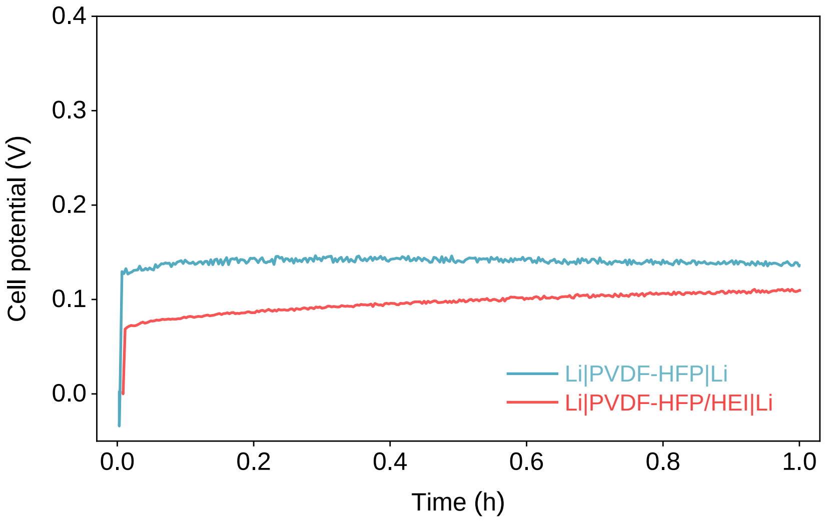

Figure S28. Lithium stripping process of the Li||Li cells with the PVDF-HFP and PVDF-HFP/HEI electrolytes, tested at 3 mA cm^{-2} and 25°C.

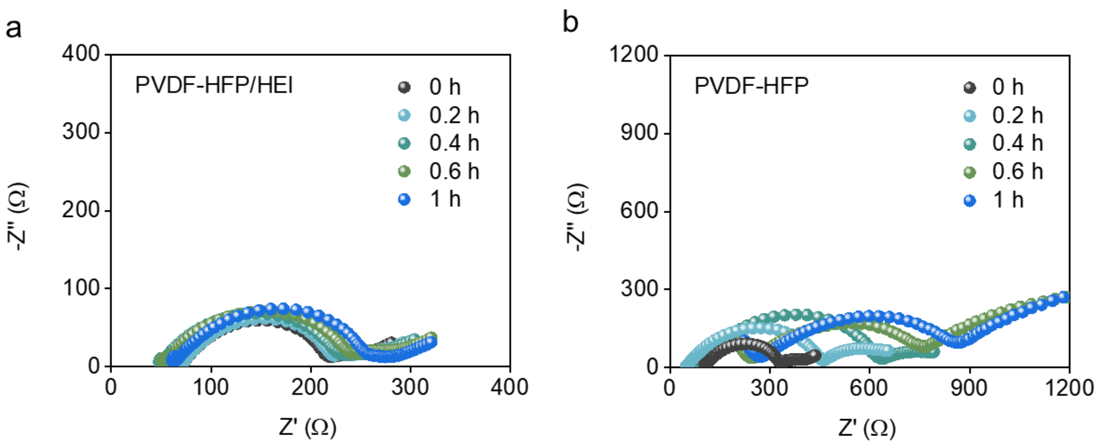

Figure S29. In-situ Nyquist plots of the symmetric Li||Li cells with PVDF-HFP/HEI (a) and PVDF-HFP electrolytes (b), tested at 3.0 mA cm^{-2} and 25°C.

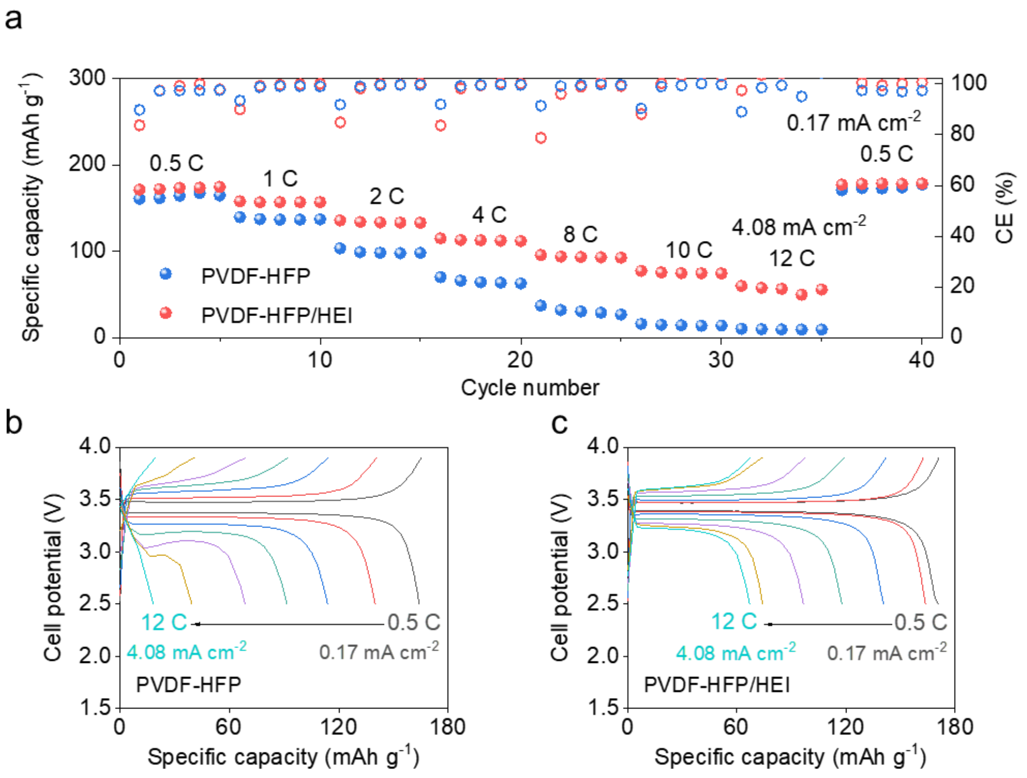

Figure S30. (a) Rate performance of Li||LiFePO$_4$ cells with PVDF-HFP and PVDF-HFP/HEI electrolytes, tested from 0.17 mA cm^{-2} to 4.08 mA cm^{-2} and 25°C. (b, c) Charge/discharge profiles of Li||LiFePO$_4$ cells with PVDF-HFP (b) and PVDF-HFP/HEI (c) electrolytes.

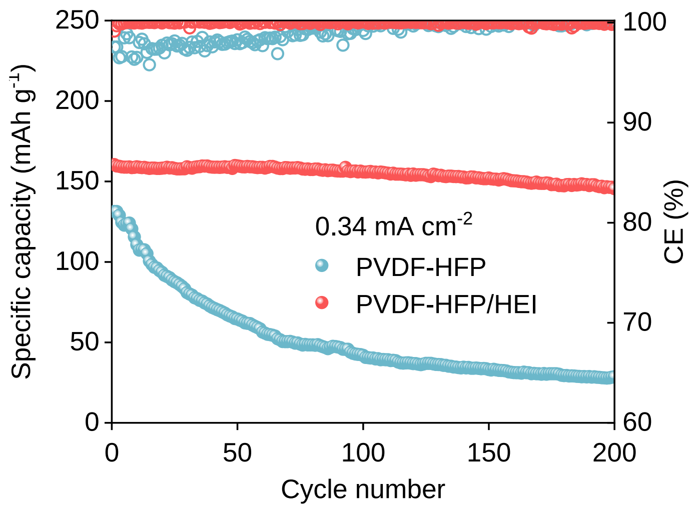

Figure S31. Long-term cycling performance of Li||$LiFePO_4$ cells with PVDF-HFP and PVDF-HFP/HEI electrolytes, tested at 0.34 mA cm^{-2} and 25°C.

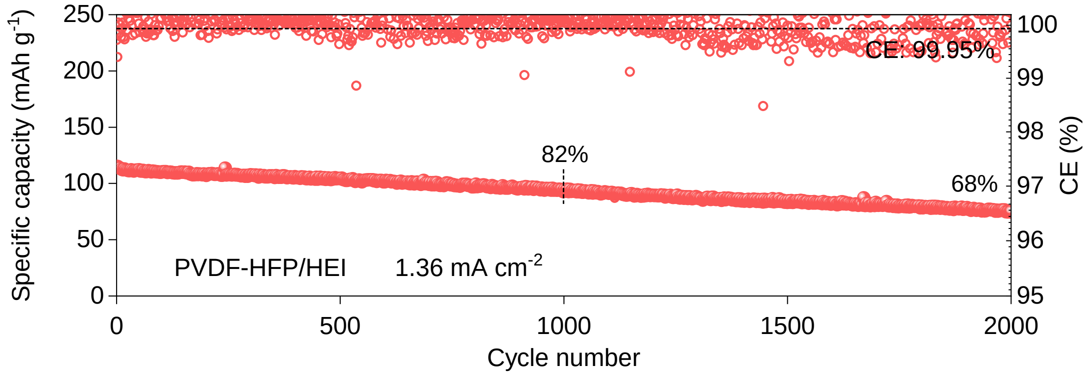

Figure S32. Long-term cycling performance of Li||$LiFePO_4$ cells with PVDF-HFP/HEI electrolyte, tested at 1.36 mA cm^{-2} and 25°C.

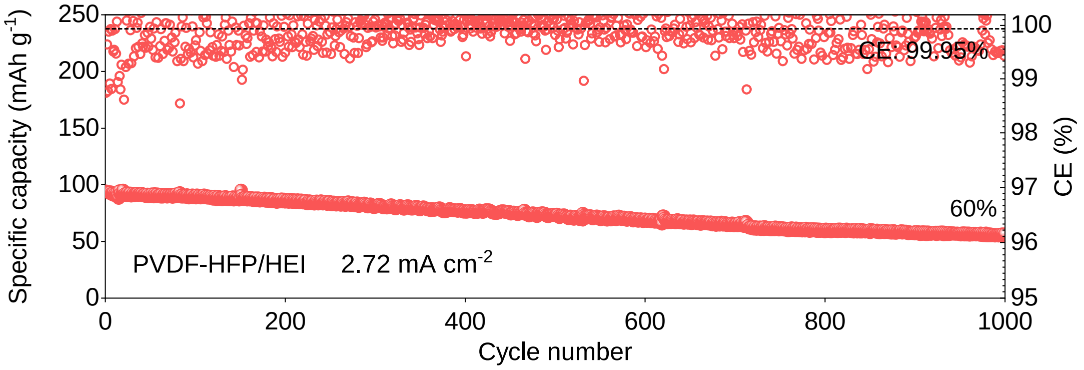

Figure S33. Long-term cycling performance of Li||$LiFePO_4$ cells with PVDF-HFP/HEI electrolyte, tested at 2.72 mA cm^{-2} and 25°C.

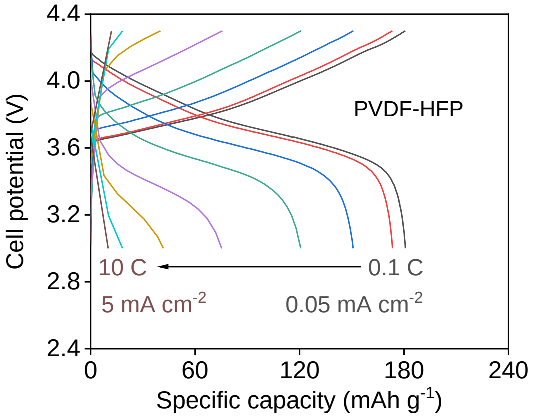

Figure S34. Charge/discharge profiles of Li||$LiNi_{0.8}Co_{0.1}Mn_{0.1}O_2$ cells with PVDF-HFP electrolyte under 0.05-5 mA cm^{-2}, corresponding to 0.1-10 C.

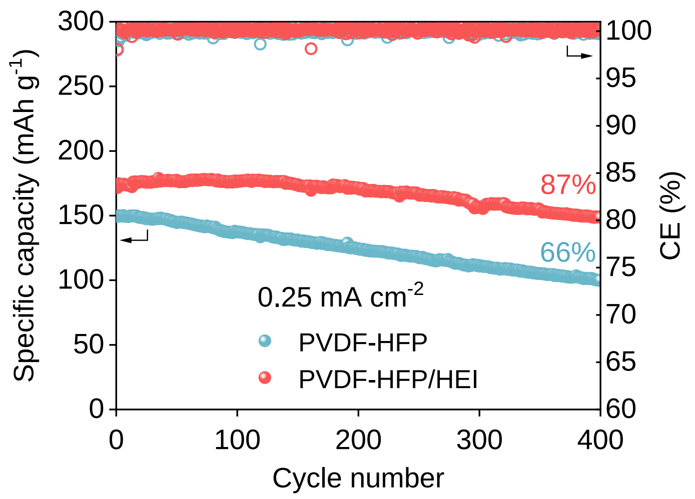

Figure S35. Long-term cycling performance of Li||$LiNi_{0.8}Co_{0.1}Mn_{0.1}O_2$ cells with PVDF-HFP and PVDF-HFP/HEI electrolytes at 0.25 mA cm^{-2}.

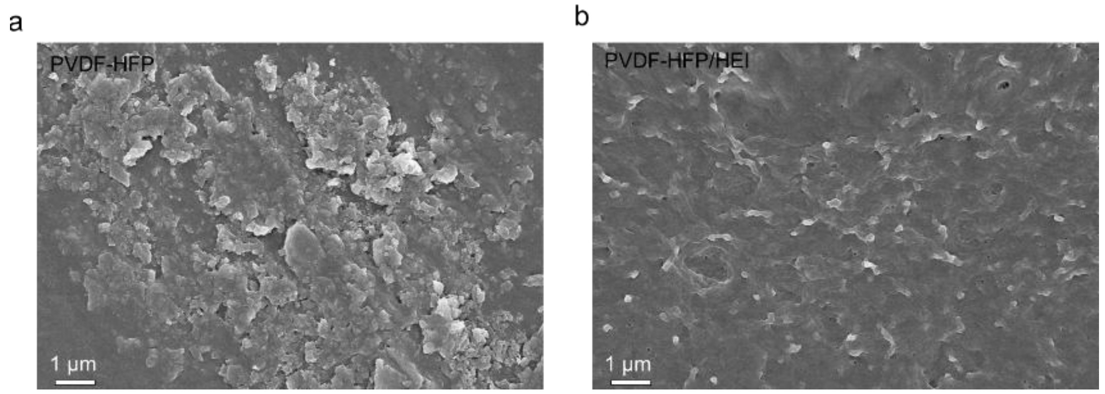

Figure S36. Surface SEM images of the PVDF-HFP electrolyte after 500 cycles (a) and PVDF-HFP/HEI electrolyte after 2000 cycles (b) in Li||$LiNi_{0.8}Co_{0.1}Mn_{0.1}O_2$ cells at 1 mA cm^{-2}.

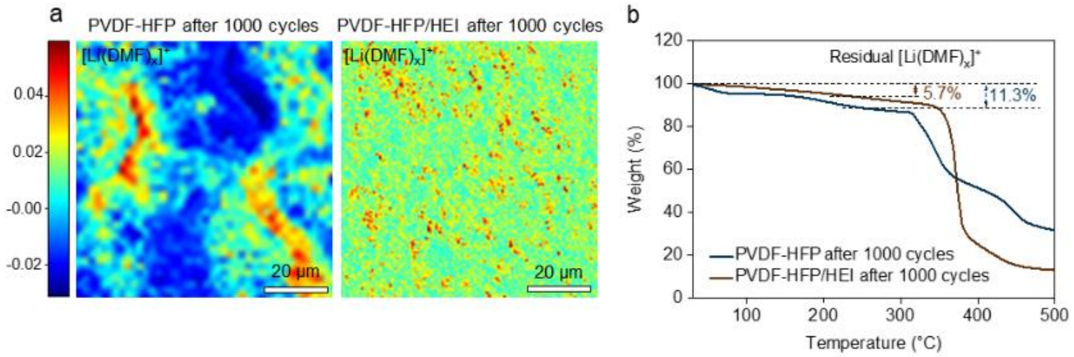

Figure S37. TOF-SIMS images showing the distribution of $[Li(DMF)_x]^+$ (a) and thermogravimetric analysis profiles (b) of PVDF-HFP and PVDF-HFP/HEI electrolytes after 1000 cycles at 3 mA cm^{-2}. The first weight-loss stage in the thermogravimetric profiles is attributed to the evaporation of $[Li(DMF)_x]^+$, while the subsequent weight loss arises from the decomposition of the polymer and lithium salt.

Table S4. The calculation basis for the energy density of Li|PVDF-HFP/HEI|$LiNi_{0.8}Co_{0.1}Mn_{0.1}O_2$ pouch cell.

**Component**		**Parameter**	
Positive electrode	$LiNi_{0.8}Co_{0.1}Mn_{0.1}O_2$	Thickness (μm)	252
		Active material loading (mg cm^{-2})	25
Negative electrode	Li foil	Thickness (μm)	20
		Areal density (mg cm^{-2})	0.85
Electrolyte	PVDF-HFP/HEI	Thickness (μm)	5
		Areal density (mg cm^{-2})	0.58
Current collector	Al foil	Thickness (μm)	10
		Areal density (mg cm^{-2})	2.7
Cell	Li|PVDF-HFP/HEI|$LiNi_{0.8}Co_{0.1}Mn_{0.1}O_2$	Areal capacity (mAh cm^{-2})	4.9
		Average discharge voltage (V)	3.73
		Toal Areal density (mg cm^{-2})	31.9
		Energy density (Wh kg^{-1})	573

Packaging excluded.

Table S5. Comparison of energy densities of solid-state pouch cells with PVDF-HFP/HEI and other reported electrolytes.

Electrolyte	Thickness of electrolyte (μm)	Cell	Energy density (Wh kg^{-1})	Reference
LLZO-PAN	16	Li|LLZO-PAN|$LiNi_{0.8}Co_{0.1}Mn_{0.1}O_2$	345.8	[14]
LLZO-MOF-polyethylene (PLM)	12.6	Li|PLM|$LiNi_{0.8}Co_{0.1}Mn_{0.1}O_2$	344	[21]
CS-CSSE	12	Li|CS-CSSE|$LiNi_{0.83}Co_{0.12}Mn_{0.05}O_2$	376	[22]
PAN-PEGDA	10	Li|PAN-PEGDA|$LiNi_{0.8}Co_{0.1}Mn_{0.1}O_2$	380	[11]
SSE@PH-BN/LN	10	Li|SSE@PH-BN/LN|$LiNi_{0.8}Co_{0.1}Mn_{0.1}O_2$	316.6	[23]
LSE-HFC	9	Li|LSE-HFC|$LiNi_{0.6}Co_{0.2}Mn_{0.2}O_2$	420.7	[24]
PPLA	8.4	Li|PPLA|$LiNi_{0.8}Co_{0.1}Mn_{0.1}O_2$	415.2	[18]
This work (PVDF-HFP/HEI)	5	Li|PVDF-HFP/HEI|$LiNi_{0.8}Co_{0.1}Mn_{0.1}O_2$	573	

Packaging excluded.

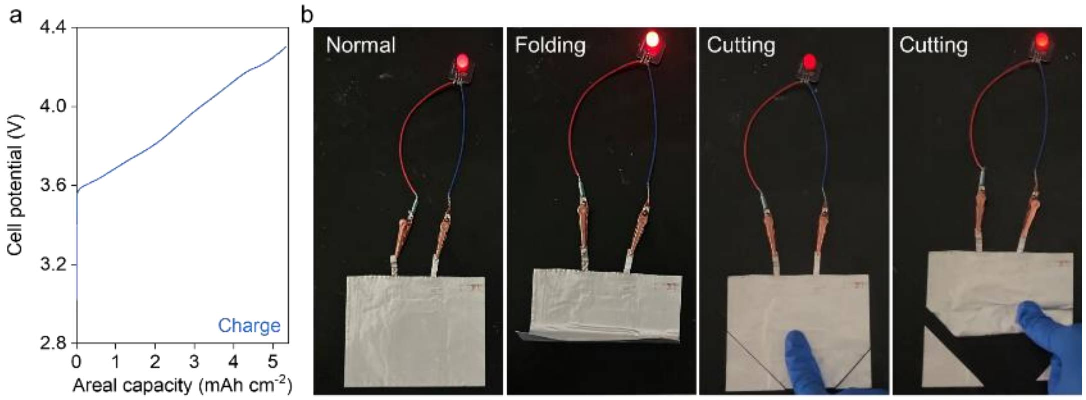

Figure S38. Safety valuation of the Li||$LiNi_{0.8}Co_{0.1}Mn_{0.1}O_2$ pouch cell with the PVDF-HFP/HEI electrolyte. (a) Charge profile of the Li||$LiNi_{0.8}Co_{0.1}Mn_{0.1}O_2$ pouch cell with the PVDF-HFP/HEI electrolyte charged to 4.3 V. (b) Photographs of the pouch cell powering a red LED under normal, folded, and cut states.

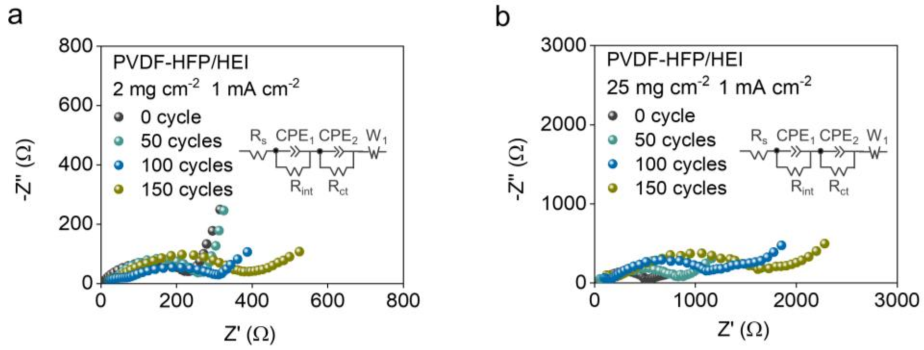

Figure S39. Nyquist plots of the Li||$LiNi_{0.8}Co_{0.1}Mn_{0.1}O_2$ cell after different cycles with $LiNi_{0.8}Co_{0.1}Mn_{0.1}O_2$ mass loading of 2 mg cm^{-2} (a) and 25 mg cm^{-2} (b).

References:

1. An XF, Liu Y, Yang K *et al.* Dielectric filler-induced hybrid interphase enabling robust solid-state Li metal batteries at high areal capacity. *Adv Mater* 2024; **36**: 2311195.
2. Tao F, Yan KJ, Dong CX *et al.* Electric-dipole coupling ion-dipole engineering induced rational solvation-desolvation behavior for constructing stable solid-state lithium metal batteries. *Angew Chem Int Ed* 2025; **64**: e202503037.
3. Li YH, Sun ZJ, Liu DY *et al.* A composite solid polymer electrolyte incorporating MnO_2 nanosheets with reinforced mechanical properties and electrochemical stability for lithium metal batteries. *J Mater Chem A* 2020; **8**: 2021-32.
4. Wu Q, Fang MD, Jiao SZ *et al.* Phase regulation enabling dense polymer-based composite electrolytes for solid-state lithium metal batteries. *Nat Commun* 2023; **14**: 6296.
5. Polu AR, Kim K, Kareem AA *et al.* Impact of tetracyanoethylene plasticizer on PEO based solid polymer electrolytes for improved ionic conductivity and solid-state lithium-ion battery performance. *J Power Sources* 2025; **625**: 235742.
6. Shi PR, Ma JB, Liu M *et al.* A dielectric electrolyte composite with high lithium-ion conductivity for high-voltage solid-state lithium metal batteries. *Nat Nanotechnol* 2023; **18**: 602-10.
7. Zhang DS, Shi YZ, An JW *et al.* Triallyl cyanurate copolymerization delivered nonflammable and fast ion conducting elastic polymer electrolytes. *J Mater Chem A* 2022; **10**: 23095-102.
8. Liu YX, Zhang DC, Luo LJ *et al.* Percolating coordinated ion transport cells in polymer electrolytes to realize room-temperature solid-state lithium metal batteries. *Energy Storage Mater* 2024; **70**: 103548.
9. Zhai PB, Yang ZL, Wei Y *et al.* Two-dimensional fluorinated graphene reinforced solid polymer electrolytes for high-performance solid-state lithium batteries. *Adv Energy Mater* 2022; **12**: 2200967.
10. Chang X, Cheng R, Wang T *et al.* Fluorinated carbon nitrides assisted solvation-regulation engineering toward polyvinylidene fluoride-based electrolytes for long-lifespan solid-state lithium batteries. *Energy Environ Sci* 2025; **18**: 9490-501.
11. Zhang YN, Yu JM, Shi HS *et al.* Fiber-reinforced ultrathin solid polymer electrolyte for solid-state lithium-metal batteries. *Adv Funct Mater* 2025; **35**: 2421054.
12. Tang WJ, Tang S, Zhang CJ *et al.* Simultaneously enhancing the thermal stability, mechanical modulus, and electrochemical performance of solid polymer electrolytes by incorporating 2D sheets. *Adv Energy Mater* 2018; **8**: 1800866.
13. Ma SQ, Zhao N, Lin J *et al.* Flexible and ultra-thin membrane electrolyte with polymer-reinforced-ceramic ion-conducting framework for sodium metal batteries. *J Energy Chem* 2025; **104**: 576-84.
14. Zhao XX, Wang C, Fan XM *et al.* Addressing the interface issues of all-solid-state lithium batteries by ultra-thin composite solid-state electrolyte combined with the integrated preparation technology. *Infomat* 2025; **7**: e70012.
15. Liu YH, Wang PH, Yang ZY *et al.* Lignin derived ultrathin all-solid polymer electrolytes with 3D single-ion nanofiber ionic bridge framework for high

performance lithium batteries. *Adv Mater* 2024; **36**: 2400970.

16. Yu FH, Mu YB, Han MS *et al.* Electrochemically stable and ultrathin polymer-based solid electrolytes for dendrite-free all-solid-state lithium-metal batteries. *Mater Futur* 2025; **4**: 015101.
17. Feng G, Ma QY, Luo D *et al.* Designing cooperative ion transport pathway in ultra-thin solid-state electrolytes toward practical lithium metal batteries. *Angew Chem Int Ed* 2025; **64**: e202413306.
18. Nie YH, Luo D, Yang TZ *et al.* Ultrathin electrolyte membranes with reinforced concrete structure for fast-charging solid-state lithium metal batteries. *Adv Mater* 2025; **37**: 2504092.
19. Ma YX, Wan JY, Yang YF *et al.* Scalable, ultrathin, and high-temperature-resistant solid polymer electrolytes for energy-dense lithium metal batteries. *Adv Energy Mater* 2022; **12**: 2103720.
20. Liu LF, Shi YZ, Liu MY *et al.* An ultrathin solid electrolyte for high-energy lithium metal batteries. *Adv Funct Mater* 2024; **34**: 2403154.
21. Zhang Z, Gou JR, Cui KX *et al.* 12.6 μm-thick asymmetric composite electrolyte with superior interfacial stability for solid-state lithium-metal batteries. *Nano-Micro Lett* 2024; **16**: 181.
22. Bao CS, Zheng CJ, Wu MF *et al.* 12 μm-thick sintered garnet ceramic skeleton enabling high-energy-density solid-state lithium metal batteries. *Adv Energy Mater* 2023; **13**: 2204028.
23. Liu KX, Xie Y, Ding T *et al.* High-strength ultrathin solid-state electrolyte with ion-rectifying honeycomb-like skeleton for lithium metal batteries free from external pressure. *Adv Funct Mater* 2026; **36**: e11558.
24. Guo SY, Su YF, Yan K *et al.* Robust and adhesive laminar solid electrolyte with homogenous and fast Li-ion conduction for high-performance all-solid-state lithium metal battery. *Adv Sci* 2024; **11**: 2404307.
